# CHCHD10 Mitigates Alzheimer's Disease‐Related Phenotypes in Association With Epigenetic Remodeling in Directly Reprogrammed Neurons

**DOI:** 10.1002/advs.76205

**Published:** 2026-06-25

**Authors:** Teresa M. Thomas, Ching‐Yao Yang, Kexin Zhang, Liam Wetzel, Dina Bugybayeva, Julia Ferguson, Md Mahmudul Hasan, William Samsa, Nandita Patil, Anshul Dash, Tarini Gowda, Xiongwei Zhu, Feixiong Cheng, Tian Liu

**Affiliations:** ^1^ Department of Pathology Case Western Reserve University School of Medicine Cleveland Ohio USA; ^2^ Cleveland Clinic Genome Center Cleveland Clinic Research Cleveland Clinic Cleveland Ohio USA; ^3^ Department of Genomic Sciences and Systems Biology Cleveland Clinic Research Cleveland Clinic Cleveland Ohio USA; ^4^ Department of Molecular Medicine Cleveland Clinic Lerner College of Medicine Case Western Reserve University Cleveland Ohio USA

**Keywords:** biology, chromatin, cpg site, dna methylation, epigenetics, epigenome, epigenomics, expression quantitative trait loci, methylation, reprogramming

## Abstract

Mitochondrial dysfunction and chromatin dysregulation are interconnected contributors to neuronal vulnerability in Alzheimer's disease (AD), yet the molecular mechanisms linking these processes remain poorly understood. CHCHD10, a mitochondrial intermembrane space protein, has been implicated in neurodegenerative disorders, but its role in AD has not been defined. Here, we identify CHCHD10 as a previously unrecognized modulator of neuronal epigenomic stability in AD. Using direct fibroblast‐to‐neuron reprogramming, which preserves patient‐specific epigenetic signatures, we show that AD neurons recapitulate genome‐wide hypomethylation patterns observed in postmortem AD cortex. CHCHD10 expression is significantly reduced in AD neurons and across multiple human brain datasets, including single‐cell and bulk RNA sequencing, proteomics, and human cortical tissue analyses. Restoration of CHCHD10 in AD neurons reduces amyloid‐β and insoluble tau accumulation while reversing AD‐associated differentially methylated regions across CpG islands, promoters, and regulatory elements. CHCHD10‐responsive methylation changes overlap with those observed in human AD brain regions and colocalize with significant AD loci and cortex‐specific eQTL loci, including MAPT and ABCA7. Finally, we identify KATNAL2 as a CHCHD10‐responsive effector whose loss enhances tau phosphorylation and seeding, whereas its restoration mitigates tau pathology. Together, these findings support a CHCHD10‐associated neuroprotective pathway linking mitochondrial dysfunction, epigenomic instability, and tau pathology in AD.

## Graphical abstract

CHCHD10 loss in Alzheimer's disease is associated with mitochondrial dysfunction, epigenomic disruption, and tau pathology. Restoration of CHCHD10 shifts DNA methylation toward a non‐disease state and reduces tau and amyloid pathology, with KATNAL2 acting as a downstream effector.

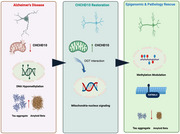

## 1 Introduction

Alzheimer's disease (AD) is a progressive neurodegenerative disorder characterized by extracellular amyloid‐β (Aβ) deposition, intracellular tau neurofibrillary tangles, synaptic degeneration, and neuronal loss [1]. Although genetic risk factors such as *APOE* and environmental exposures modulate AD susceptibility [2], accumulating evidence highlights mitochondrial dysfunction [3], impaired stress responses [4, 5], and global epigenomic disruption [6] as critical drivers of neuronal vulnerability. Mitochondria–nucleus signaling has emerged as a determinant of chromatin state and transcriptional homeostasis [7, 8], yet the pathways coupling mitochondrial stress to epigenetic remodeling remain largely unknown. While Aβ accumulation and tau aggregation, mitochondrial and mitochondria‐associated abnormalities are among the primary characteristics of Aβ pathogenesis and tauopathies [3, 9, 10]. The extensive interactions between tau and mitochondrial proteins have been confirmed in human brains and neurons [11, 12]. Targeting mitochondrial proteins represents a potentially effective approach for mitigating AD pathology [13, 14, 15].

CHCHD10 (Coiled‐Coil‐Helix‐Coiled‐Coil‐Helix Domain–Containing 10) is a mitochondrial intermembrane space protein essential for cristae architecture, oxidative phosphorylation, mitochondrial RNA processing, and cellular stress adaptation [16]. Pathogenic CHCHD10 variants cause multi‐systemic proteinopathy, frontotemporal dementia (FTD), amyotrophic lateral sclerosis (ALS), and mitochondrial myopathy [17, 18, 19, 20, 21]. These disorders share pathological features with AD, including mitochondrial fragmentation, oxidative stress, protein aggregation, and altered RNA metabolism [22, 23, 24, 25]. However, whether CHCHD10 contributes to AD pathogenesis and whether it interfaces with the epigenetic landscape remains unknown.

Epigenetic dysregulation, particularly DNA methylation changes, is an emerging hallmark of AD [26, 27]. Methylation is one of the most well‐studied epigenetic modifications of the genome. Typically occurring at the 5‐cytosine location on 5'—C—phosphate—G—3' (CpG) dinucleotides, creating 5‐methylcytosines (5mC) [28]. Many CpG dinucleotides are clustered in structures called CpG islands, which are often associated with gene promoters [29]. The methylation of DNA is known to be involved in gene and chromatin regulation, as well as important biological processes, from imprinting to X chromosome inactivation to gametogenesis [30]. Methylation changes, both hypermethylation and hypomethylation, have been associated with multiple neurological disorders, including AD [31]. Recent AD studies have established the loss of methylated cytosine in both neocortical and hippocampal neurons [32]. Studies consistently demonstrate widespread hypomethylation in cortical neurons, affecting promoters, enhancers, and transcription factor binding sites. AD neurons often exhibit global hypomethylation, particularly in CpG islands and promoters, which may result in aberrant gene activation [33, 34, 35]. Hypomethylation in AD brains has been shown to alter transcription and influence the pathology of AD [36, 37]. Methylation alterations correlate with Aβ burden, tau pathology, cognitive decline, and selective neuronal vulnerability [33, 38]. However, the upstream drivers of these epigenetic changes are incompletely understood, partly due to the difficulty in modeling age‐associated epigenomic patterns in vitro.

**FIGURE 1:**
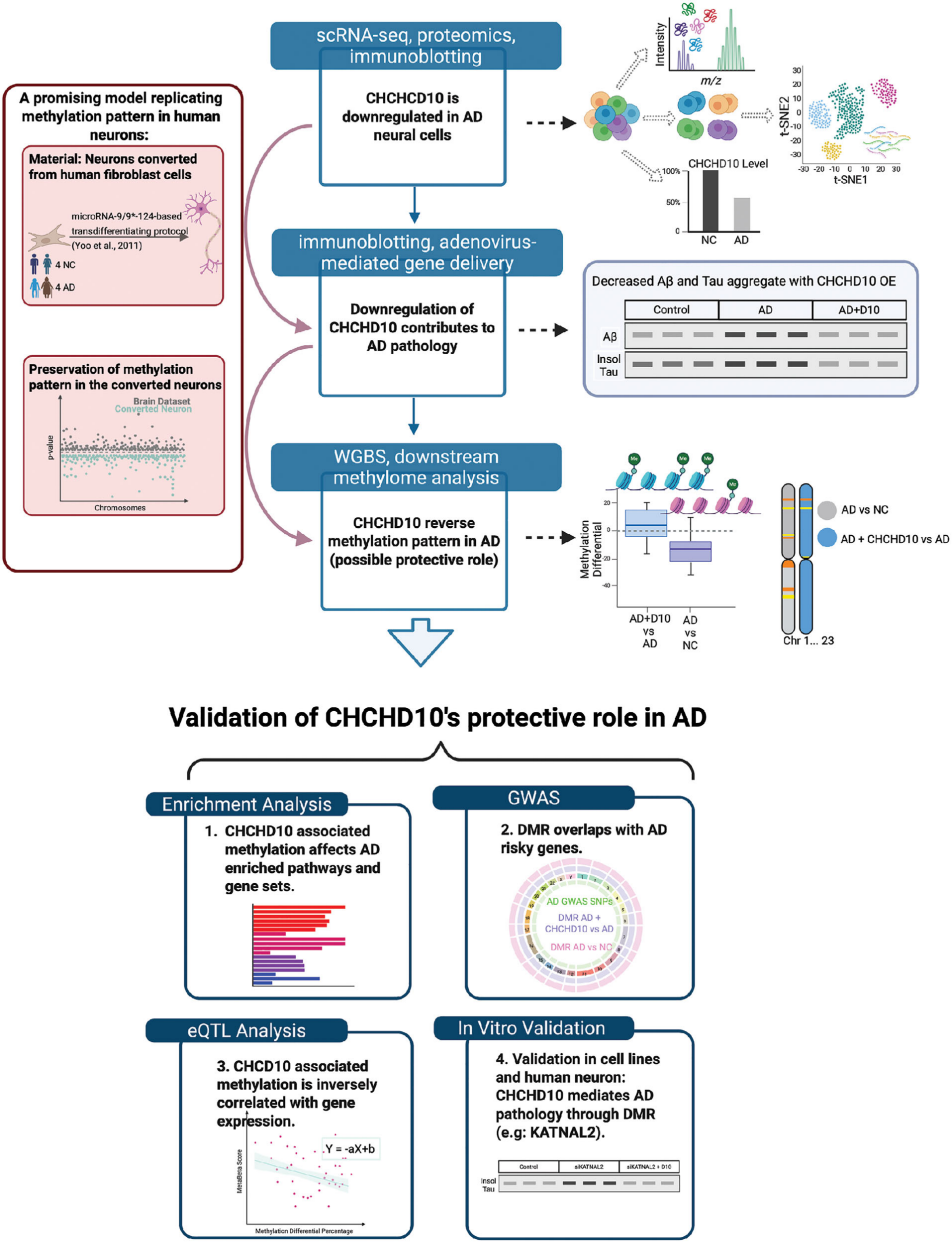
Abstract Illustration: Framework of CHCHD10‐associated DNA methylation analysis in AD. This schematic outlines the major experimental and analytical steps in the study, including the generation of fibroblast‐reprogrammed neurons, the assessment of CHCHD10 expression, multi‐omics profiling (DNA methylation, RNA‐seq, proteomics), comparisons with human AD brain datasets, and functional assays involving CHCHD10 overexpression and KATNAL2 manipulation. Downstream analyses include differential methylation testing, integrative correlation analyses with AD pathology, and mechanistic validation in cell lines and neuron‐based models.

To overcome these challenges, we utilized microRNA‐9/9*‐124‐mediated direct reprogramming of human fibroblasts into neurons (Figure 1) [39]. Neurons by direct reprogramming allow for the retention of epigenomic signatures relevant for studying age‐ and disease‐associated methylation changes [39, 40, 41, 42], making them a powerful system for studying epigenetic contributors to neurodegeneration. Using this model, we found that CHCHD10 is markedly reduced in AD neurons and human AD cortex at transcript and protein levels (Figure 1). Restoration of CHCHD10 reversed pathogenic Aβ and tau phenotypes and was associated with widespread remodeling of AD‐associated DNA methylation, including reversal of hypomethylated and hypermethylated DMRs that overlapped with alterations in the human brain (Figure 1). We also integrated whole‐genome bisulfite sequencing (WGBS) data with large‐scale AD GWAS and cortex‐specific eQTL datasets to test whether CHCHD10‐responsive methylomic changes intersect with genetic risk architecture (Figure 1). We identified loci with strong colocalization, including MAPT, ABCA7, and KANSL1, suggesting that CHCHD10 may regulate methylation at genetically vulnerable regions. Among several candidate genes emerging from DMR and eQTL‐methylation modeling, KATNAL2, encoding a microtubule‐severing protein, was consistently associated with AD‐linked hypomethylation that was shifted toward NC‐like methylation patterns by CHCHD10 overexpression in reprogrammed neurons. Functional assays demonstrated that KATNAL2 reduction increases tau phosphorylation and seeding activity, whereas CHCHD10 and KATNAL2 overexpression mitigate tau pathology.

In this study, we reveal CHCHD10 as a previously unrecognized modulator of the neuronal epigenome in AD and identify the CHCHD10–KATNAL2 association that functionally modulates tau pathology. These findings bridge mitochondrial dysfunction and epigenetic dysregulation, providing insight into early pathogenic events and nominating CHCHD10 as a therapeutic target for restoring chromatin homeostasis and attenuating neurodegeneration.

## 2 Results

### 2.1 CHCHD10 Expression Is Reduced in AD and Correlates With Neuropathology

**FIGURE 2:**
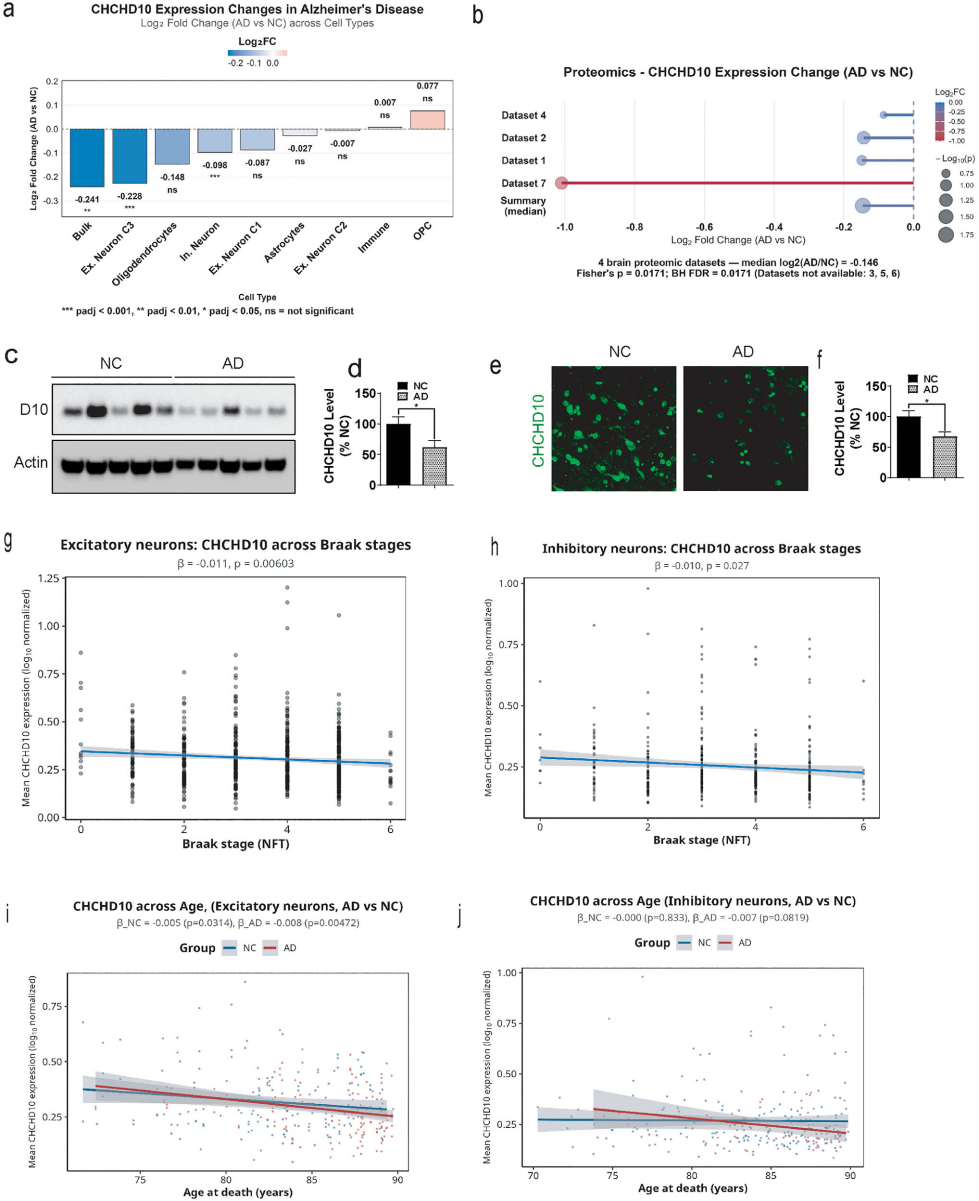
Downregulation of CHCHD10 in AD and its potential role. (a) CHCHD10 expression in cell types of inhibitory neuron, 3 clusters of excitatory neuron, oligodendrocyte, oligodendrocyte precursor cells (OPC), astrocyte, and immune cells of human cortex scRNA‐seq (NC n = 292, AD n = 135) and bulk RNA‐seq (NC n = 78, AD n = 82). Significance determined by p‐adjusted values < 0.05. (b) Proteomic quantification from the meta‐analysis of seven TMT datasets in the *Proteomic landscape of AD* (44) demonstrated significantly decreased CHCHD10 abundance in AD relative to NC (log_2_[AD/NC] = −0.146; p = 0.0171; FDR = 0.0171). (c) Representative immunoblots of CHCHD10 from RIPA‐soluble lysates of human cortex (NC n = 10, AD n = 10). *Note*: Actin loading controls shown here are shared with Figure 7b because CHCHD10 and KATNAL2 were analyzed from the same brain lysates and immunoblot experiment. (d) CHCHD10 protein quantification; unpaired *t*‐test p‐value = 0.0266. (e) Immunofluorescence staining of CHCHD10 in human cortex tissue. (f) Quantification of CHCHD10 staining intensity (unpaired *t*‐test, *p*‐value = 0.0112, NC n = 5, AD n = 5). (g) Excitatory neurons (C1–C3 pooled), ROSMAP scRNA‐seq: CHCHD10 vs. Braak; linear fit β = −0.011, *p* = 0.0060. (i) Excitatory neurons (C1–C3 pooled): CHCHD10 vs. age; separate NC and AD fits (β_NC = −0.005, *p* = 0.031; β_AD = −0.008, *p* = 0.0047). (h) Inhibitory neurons: CHCHD10 vs. Braak; β = −0.010, *p* = 0.027. (j) Inhibitory neurons: CHCHD10 vs. age; β_NC ≈ 0, *p* = 0.833; β_AD = −0.007, *p* = 0.0819. Points show per‐donor means (log10‐normalized), shading = 95% CI for the OLS fit.

Mitochondrial protein CHCHD10 has been implicated in several neurodegenerative diseases [17, 20, 43], but its specific involvement in AD remains unclear. To evaluate whether CHCHD10 is altered in AD, we first examined its expression across multiple human datasets. After quality control and integration of 135 AD and 292 NC donors, CHCHD10 transcripts were markedly decreased in L5–L6 RORB‐enriched excitatory neurons, as well as in inhibitory neurons, relative to NC (Figure 2a). Bulk RNA‐sequencing from 82 AD and 78 NC temporal cortex samples similarly demonstrated reduced CHCHD10 expression in AD (Figure 2a). We next validated these transcriptomic patterns at the protein level. Re‐analysis of the Proteomic Landscape of AD (meta‐analysis of seven TMT datasets) [44] showed significantly decreased CHCHD10 protein abundance in AD cortex (log_2_[AD/NC] = −0.146, p = 0.0171, FDR = 0.0171) (Figure 2b). Immunoblotting of human cortical lysates confirmed this reduction (Figure 2c,d). Immunohistochemistry further revealed diminished CHCHD10 immunofluorescence in AD cortical neurons (Figure 2e,f). These findings corroborate the transcriptomic and tissue‐level data, confirming CHCHD10 loss as a reproducible molecular feature of AD pathology.

We next assessed if CHCHD10 expression tracked AD neuropathology in human neurons. Using ROSMAP scRNA‐seq, we next related per‐donor mean CHCHD10 expression to Braak stage and age at death within neurons. In both excitatory and inhibitory neurons (aggregating clusters C1–C3), pooled linear models (no covariates) showed a significant negative association with Braak (β = −0.011, *p* = 0.0060 in excitatory neurons; β = −0.010, *p* = 0.027 in inhibitory neurons) (Figure 2g,h). We also observed a negative association between excitatory and inhibitory CHCHD10 with age in both NC (β = −0.005, *p* = 0.031) and AD (β = −0.008, *p* = 0.0047) groups (Figure 2i,j). The age trend was directionally negative and nominal in AD (β = −0.007, *p* = 0.082) but not in NC (β ≈ 0, *p* = 0.83). Together, these multi‐modal human data demonstrate that CHCHD10 reduction is a reproducible molecular signature of AD and correlates with tau pathology and age‐related neuronal vulnerability.

### 2.2 Restoring CHCHD10 in Reprogrammed AD Neurons Reduces Ab and Tau Pathology

**FIGURE 3:**
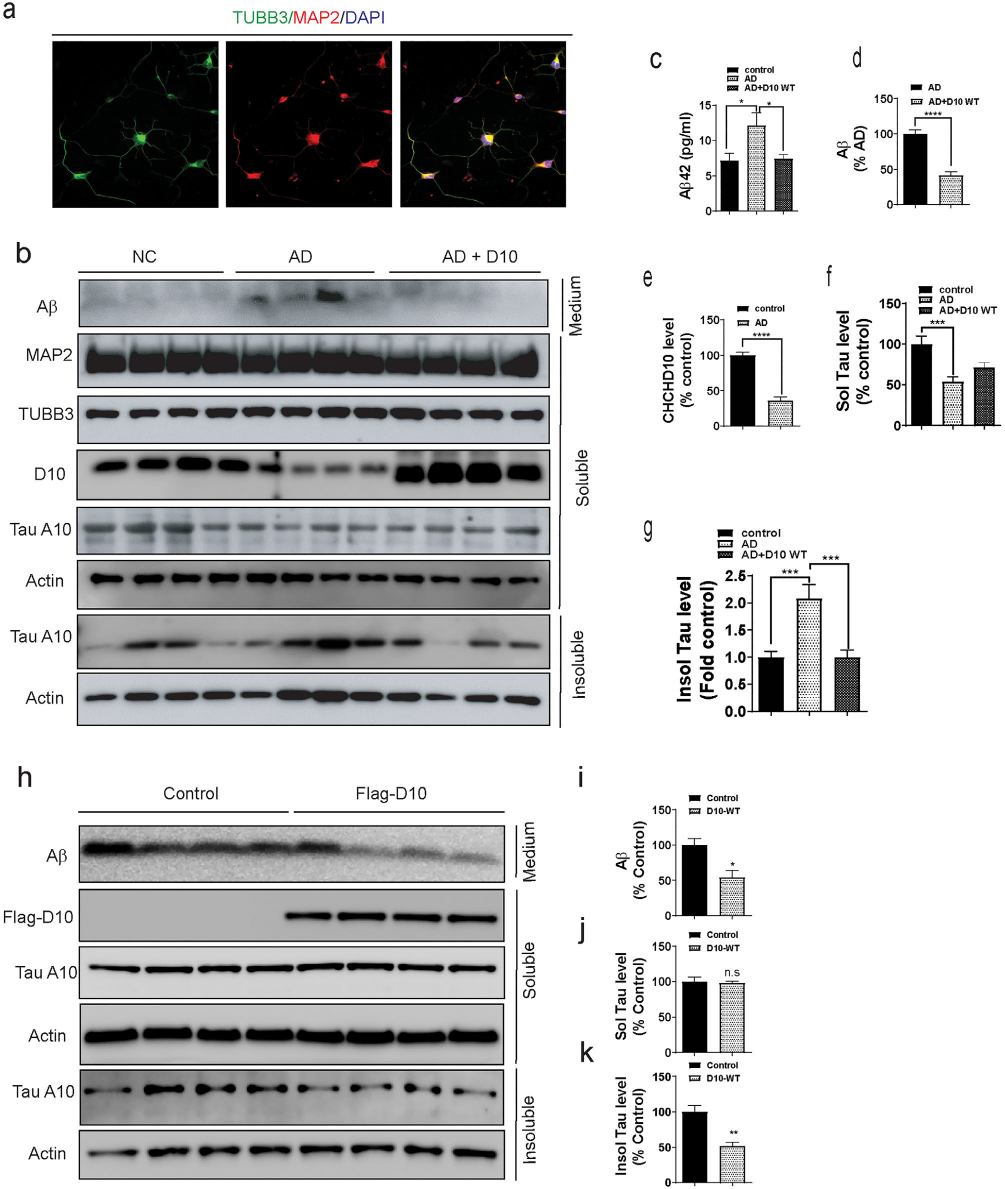
CHCHD10 overexpression reduces Aβ production and tau aggregation in AD neurons. (a) Representative images for the primary fibroblasts‐converted neurons stained for MAP2, TUBB3, and DAPI. (b) Representative immunoblots of Aβ, CHCHD10, Tau, TUBB3, MAP2, and actin in converted neurons from normal control, AD, and AD+CHCHD10WT groups (n = 4 for each group). AD neurons were transduced by the CHCHD10 adenovirus at day 21 for 7 days. (c–g) Quantifications of protein expression levels from normal control, AD, and AD+CHCHD10WT groups. (c) Aβ42 level measured by ELISA Ab42 kit (one‐way ANOVA Sidak's test, ^*^ *p* < 0.05). (d) Aβ levels using the Aβ antibody. (e) CHCHD10 protein levels (unpaired t‐test p‐value < 0.0001); (f) RIPA‐soluble Tau levels (one‐way ANOVA Sidak's test, Control vs. AD p‐value = 0.0005); (g) RIPA‐insoluble Tau levels (one‐way ANOVA Sidak's test p‐values = 0.006 for Control vs. AD and 0.006 AD vs. AD+CHCHD10WT). (h) Representative immunoblots of Aβ, Flag‐CHCHD10, Tau, CHCHD10, Tau, and actin from medium samples or RIPA‐soluble and insoluble fractions of M17‐APP stable cells transfected with control vector and p3x‐Flag‐CHCHD10. (i‐k) Quantifications of protein expression levels of Aβ and tau (n = 4 for each group, unpaired *t*‐test, ^*^ *p*‐value = 0.0148, ^**^ *p*‐value = 0.0036, n.s: not significant).

To examine whether CHCHD10 loss contributes to AD‐related phenotypes, we used microRNA‐9/9*‐124‐based direct reprogramming to generate neurons from primary fibroblasts of four AD and four NC donors. These neurons retain donor‐specific epigenomic signatures while acquiring mature neuronal markers (Figure 3a). Interestingly, overexpression of CHCHD10 in AD neurons was achieved by adenoviral transduction on day 21 of differentiation, with analyses performed on day 28 (Figure 3b). AD neurons exhibited significantly increased Aβ production relative to NC neurons, as measured by ELISA and immunoblotting (Figure 3b–d). Total CHCHD10 protein level was significantly decreased in AD neurons when compared to controls (Figure 3e). Tau pathology was next assessed in soluble and insoluble fractions. AD neurons demonstrated elevated soluble tau (RIPA fraction) and markedly increased insoluble tau compared to NC (Figure 3f,g). Strikingly, CHCHD10 overexpression selectively decreased insoluble tau levels, restoring them toward the NC baseline (Figure 3g). Consistent with these findings, CHCHD10 overexpression in human neuroblastoma M17‐APP stable cells significantly reduced Aβ and insoluble tau levels (Figure 3h–k), as well as both soluble and insoluble pTau231 levels (Figure S1b, c). In contrast, CHCHD10 knockdown in M17‐APP cells increased insoluble tau and both soluble and insoluble pTau231 levels (Figure S1d, e). These findings demonstrate that CHCHD10 downregulation could be a contributing factor to AD neuronal phenotypes and that restoring CHCHD10 could mitigate both amyloid and tau pathology.

### 2.3 CHCHD10 Overexpression Reverses AD‐Associated DNA Methylation Abnormalities

In AD, aberrant methylation contributes to dysregulation of neuronal genes involved in inflammation, protein turnover, and synaptic function [45, 46, 47]. Modeling epigenetic features of AD in vitro requires validation that cellular systems faithfully reproduce brain‐specific methylation patterns. To investigate whether CHCHD10 influences the methylome, we performed whole‐genome bisulfite sequencing (WGBS) on reprogrammed neurons grouped as NC, AD, and AD+CHCHD10. Differentially methylated regions (DMRs) identified in our neuron‐derived AD vs. NC dataset were analyzed. In comparison with DMR derived from AD vs. NC brains, DMR between AD and NC neurons significantly overlapped with DMRs identified in human postmortem cortex, Middle temporal gyrus (MTG) (p = 3.789 × 10^−4^; Figure S2a) and DMRs in the prefrontal cortex (PFC) (p = 1.002 × 10^−2^; Figure S2b). These results confirm that direct‐reprogrammed neurons faithfully retain clinically relevant methylomic patterns. We further investigated the overlap between genes near DMRs in our aggregated AD + CHCHD10 vs. AD dataset (including both CpG and promoter regions) and the brain PFC AD vs. NC dataset. Using significant DMRs, Fisher's exact test yielded a p‐value of 0.037 (Figure S3a), indicating a modest but significant overlap. In contrast, no significant overlap was found between our AD + CHCHD10 vs. AD dataset and the brain MTG dataset (p = 0.088; Figure Sb). These findings suggest that CHCHD10 overexpression in AD neurons alters methylation at gene loci that partially intersect with native brain methylation patterns observed in AD.

### 2.4 CHCHD10 Overexpression Reverses Disease‐Associated Methylation Alterations

**FIGURE 4:**
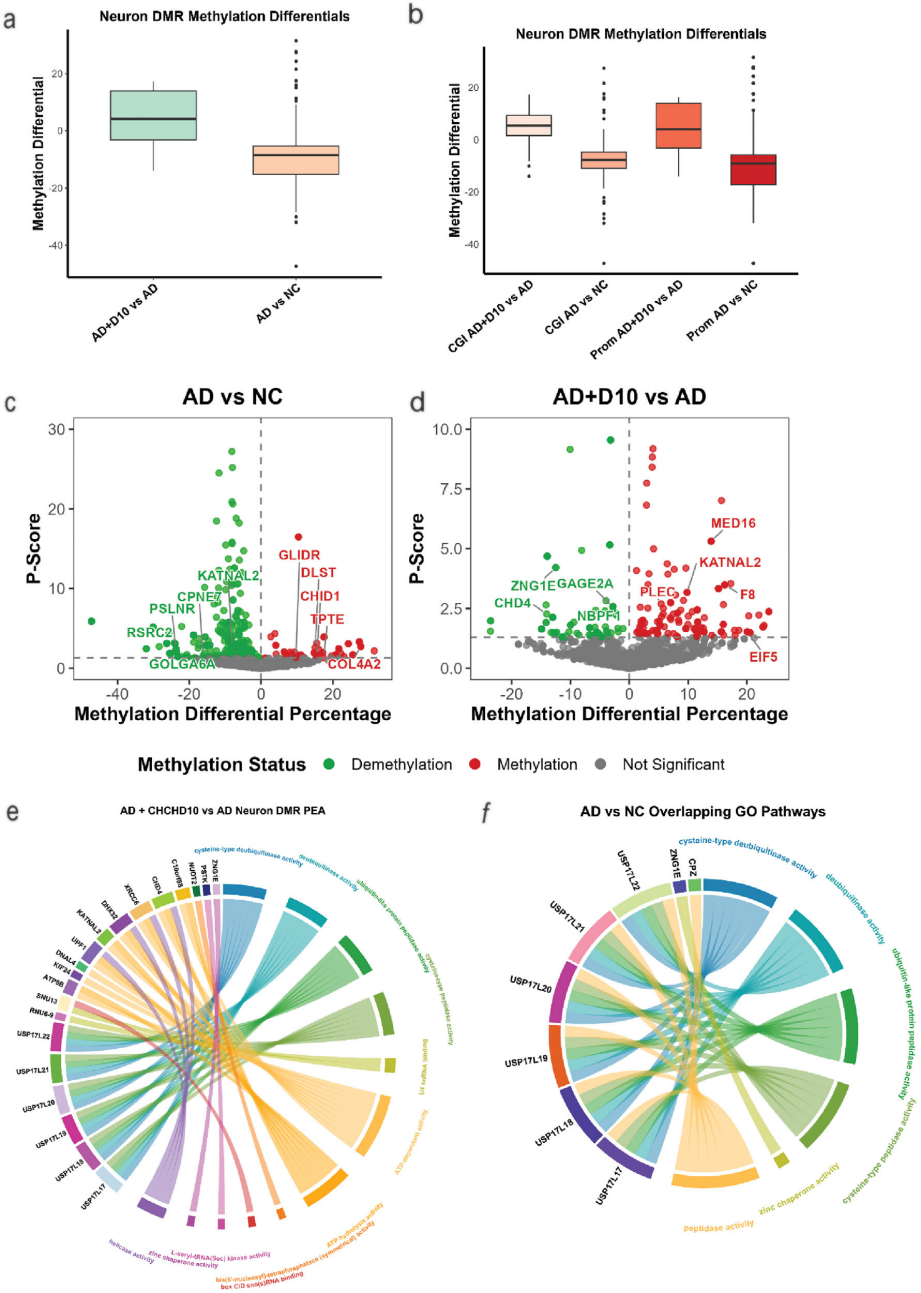
CHCHD10 overexpression reverses DNA methylation changes in AD neurons. (a) Boxplot of methylation differential percentages (q‐value < 0.05) in aggregate datasets. Two‐tailed *t*‐test p‐value = 2.718 × 10^−14^; comparison of means p‐value < 0.0001. (b) Boxplot of significant DMRs in CGI and promoter datasets. CGI: two‐tailed t‐test p‐value = 1.95 × 10^−5^; the comparison of means p‐value = 0.0001. Promoter: two‐tailed t‐test p‐value = 3.76 × 10^−10^; comparison of means p‐value < 0.0001. (c, d) Volcano plots of significant (*p*‐value < 0.05) DMRs in AD vs. NC and AD+CHCHD10 vs. AD datasets. (e) Chord diagram of the top 12 enriched GO molecular function pathways (*p*‐value < 0.05) from AD+CHCHD10 vs. AD DMRs. (f) Chord diagram of 6 overlapping GO molecular function pathways (*p*‐value < 0.05) in AD vs. NC and AD+CHCHD10 vs. AD datasets.

After validating the significance of the AD vs. NC DMR results, we next sought to characterize the overall directionality and magnitude of methylation changes within these regions. For this analysis, we used significant DMRs (q‐value < 0.05) from the aggregated AD vs. NC and AD + CHCHD10 vs. AD datasets (Figure 4a). Methylation differential percentages were computed for each DMR. The mean differential in the AD vs. NC dataset was −9.066% (Figure 4a), indicating hypomethylation, while the AD + CHCHD10 vs. AD dataset showed a mean differential of 4.137% (Figure 4a), indicating hypermethylation. An independent two‐tailed t‐test assuming unequal variances yielded a highly significant p‐value of 2.718 × 10^−^^14^ with a t‐statistic of −8.413 (Figure 4a). A complementary comparison of means test produced a p‐value < 0.0001 with a t‐statistic ranging from 9.2065 to 17.1995 (Figure 4a). We applied the same methodology to CpG island DMRs. The average methylation differential in CpG regions was −7.423% in the AD vs. NC dataset and 4.531% in the AD + CHCHD10 vs. AD dataset (Figure 4b). This comparison yielded a t‐test p‐value of 1.954 × 10^−^^5^ and a t‐statistic of −4.914. The comparison of means test confirmed the difference with a p‐value of 0.0001 (Figure 4b). Similarly, for promoter regions, the AD vs. NC dataset showed a mean differential of −9.785%, whereas the AD + CHCHD10 vs. AD dataset had a mean differential of 3.966% (Figure 4b). The two‐tailed *t*‐test produced a p‐value of 3.763 × 10^−^^10^ and a t‐statistic of −6.884; the comparison of means test yielded a p‐value < 0.0001 (Figure 4b). Further analysis revealed that in the AD vs. NC aggregated dataset, 84.77% (206/243) of significant DMRs were associated with decreased methylation. Conversely, in the AD + CHCHD10 vs. AD dataset, 69.84% (44/63) of significant DMRs were associated with increased methylation (Figure 4c,d). Thus, across all three data types—aggregated, CpG island, and promoter DMRs—CHCHD10 overexpression reversed the direction of methylation changes observed in AD vs. NC comparisons.

To explore the functional implications of these methylation reversals, we investigated the genomic distribution and gene‐level associations of significant DMRs from the aggregate datasets. We computed the frequency of significant DMRs on each chromosome for both comparisons (Figure S1). To further visualize the methylation shifts and identify genes harboring significant DMRs, volcano plots were generated, displaying both the magnitude and direction of methylation changes, alongside representative genes linked to these regions (Figure 4c,d). Taken together, CHCHD10 overexpression is associated with a reduction in AD‐related epigenomic disruption, with methylation patterns trending toward those observed in NC.

To investigate potential mechanisms underlying CHCHD10‐mediated restoration of DNA methylation, we first assessed its subcellular localization. In addition to its known mitochondrial localization, CHCHD10 exhibited partial nuclear localization in both M17‐APP cells and human AD neurons (Figure S4a,b), supporting the possibility of direct involvement in nuclear regulatory processes. We next examined the CHCHD10 protein interaction network using immunoprecipitation‐based proteomics from APP/PS1;CHCHD10 transgenic mouse cortical brains, which identified an interaction with OGT, a chromatin‐associated regulator implicated in epigenetic processes [30, 48] (Figure S4c). This interaction was further supported by proximity ligation assays in both APP/PS1;CHCHD10 transgenic mouse brain and HT22 cells (Figure S4d–f), suggesting a close association between CHCHD10 and OGT in situ. Together, these findings suggest that CHCHD10 may be associated with OGT‐linked epigenetic regulatory pathways, although direct control of DNA methylation remains to be established.

### 2.5 Enrichment of AD‐Associated Pathways and Phenotypes in CHCHD10‐Mediated DMRs

To better understand the functional implications of DMR‐associated genes and potential mechanisms affected by CHCHD10‐mediated methylation changes, we conducted a pathway enrichment analysis. Genes located within 25 kb of each significant DMR (p < 0.05) in the aggregated AD + CHCHD10 vs. AD dataset were analyzed using the Gene Ontology (GO) Molecular Function dataset. This analysis identified 62 significantly enriched pathways (p < 0.05; Table S6), including ATPase activity, ubiquitin‐related enzymatic functions, and small nuclear RNA (snRNA) binding. These pathways overlap with known mechanisms underlying AD pathology, including proteostasis, RNA splicing, and mitochondrial function (Figure 4e). To assess whether these pathways were unique to CHCHD10 expression or also implicated in AD pathology more broadly, we performed a similar GO enrichment analysis on the AD vs. NC aggregated dataset. This analysis identified 25 significantly enriched pathways, 6 of which overlapped with the AD + CHCHD10 vs. AD dataset (Figure 4f and Table S7). These included ubiquitin‐dependent proteolysis and cysteine‐type peptidase activity—both associated with tau and Aβ turnover [49, 50, 51, 52, 53].

To further explore the phenotypic relevance of these findings, we performed gene set enrichment analysis using the Human Phenotype Ontology (HPO) via the Metabrain Network enrichment tool (https://network.metabrain.nl/gene‐list/). We identified that AD+CHCHD10 DMRs were enriched for 270 HPO terms, including abnormal nervous system morphology, gliosis, motor axonal neuropathy, and inflammatory response abnormalities (Table S8). Ninety‐two HPO terms overlapped with those found in the AD + CHCHD10 vs. AD dataset. These findings indicate that CHCHD10‐responsive methylation targets genes involved in synaptic, cytoskeletal, and neuroinflammatory pathways central to AD pathobiology.

### 2.6 Co‐Methylation Network Analysis Identifies Regulatory Modules Affected by CHCHD10

While pathway and phenotype enrichment analyses provide insights into biological processes potentially impacted by differential methylation, we sought to gain a deeper understanding of the underlying methylation patterns. To this end, we constructed a co‐methylation network to identify potential methylation motifs and uncover regions co‐regulated by shared epigenetic mechanisms (Figure S4f). Network clustering identified eight distinct communities. Five hub regions corresponding to LSP1P4, NUDT2, KIF24, and SNU13 acted as key connectors between communities (Figure S4g). These hubs are involved in RNA processing, microtubule regulation, and metabolic enzyme activity (Table S5 and Figure S4g). The presence of spliceosome‐related hubs (e.g., SNU13) suggests coordinated epigenetic modulation of RNA processing pathways, consistent with their involvement in tauopathies and mitochondrial dysfunction [54, 55, 56].

### 2.7 Regionality of AD‐Associated Methylation Reversals with CHCHD10 Expression

**FIGURE 5:**
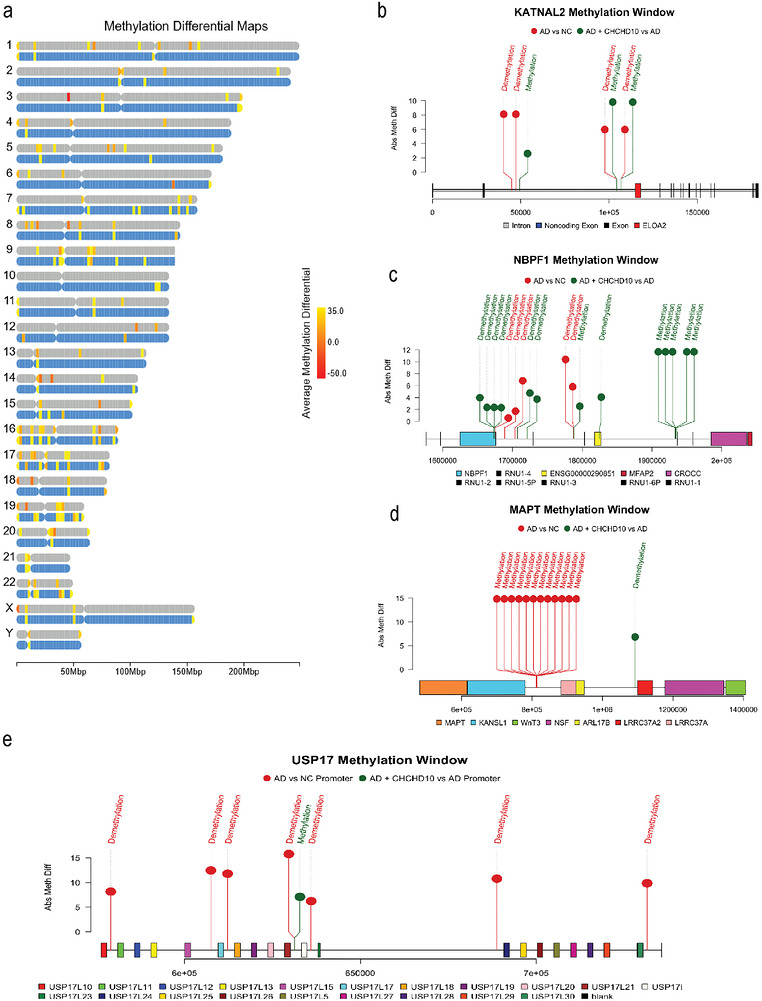
Genome‐wide DMR distribution and case gene methylation patterns. (a) Chromomap of average methylation differentials in AD vs. NC (gray) and AD+CHCHD10 vs. AD (blue); significance threshold *p*‐value < 0.05. (b–e) Lollipop plots of significant DMRs (*p*‐value < 0.05) for KATNAL2, NBPF1, MAPT, and USP17L.

DNA methylation can influence gene expression across long genomic distances, often extending beyond the loci directly harboring DMRs [57, 58, 59]. To gain a broader perspective on the genomic distribution of methylation changes, we generated a chromomap visualization to display the average methylation differential percentages of significant DMRs across scaled genomic windows (Figure 5a). This comparative analysis between the aggregated AD vs. NC and AD + CHCHD10 vs. AD datasets identified 40 overlapping genomic windows with differential methylation signatures (Figure 5a). While the chromomap provides a global view of shared methylation alterations, we further performed locus‐focused analyses to interrogate representative regions in greater detail (Figure 5b–e and Figure S5a–c). Candidate regions were selected based on the magnitude of methylation change across conditions and the presence of genes with potential relevance to neurodegeneration within affected windows. Notably, these regions encompassed KATNAL2 (Figure 5b, detailed ENCODE 4 Cis‐Regulatory Elements (cCREs) and CTCF‐associated annotation in Figure S5a), NBPF1 (Figure 5c), MAPT (Figure 5d), and USP17L family genes (Figure 5e), as well as additional loci including ZNG1E (Figure S5b), SHROOM2 (Figure S5c), and multiple regions on chromosome 19 (Figure S5d). Thus, these locus‐level examples illustrate that CHCHD10‐dependent methylation remodeling extends across broad genomic domains and preferentially involves genes within networks implicated in neurodegeneration, highlighting specific regions for future functional investigation.

### 2.8 CHCHD10‐Mediated DMRs Overlap With AD Genetic Risk Architecture

Elucidating the relationship between epigenetic modifications and genetic variation remains a central challenge in neurogenomics. To interrogate this relationship, we first performed a genome‐wide colocalization analysis using top cis‐eQTL effects from the EUR cortex dataset and AD GWAS SNPs. Analysis of 16,779 SNPs yielded posterior probabilities for hypotheses H0–H4 of 2.96 × 10^−^^7^ ^2^, 1.95 × 10^−^^4^ ^6^, 1.52 × 10^−^^2^ ^6^, 1.00, and 1.34 × 10^−^^2^ ^3^, respectively (Table S10), indicating limited support for shared causal variants (H4). Consistent with this, SNP priors were small (P1 = 5.96 × 10^−^^5^, P2 = 5.96 × 10^−^^5^, P12 = 1.00 × 10^−^^5^), suggesting that the limited genome coverage of top‐effect cis‐eQTLs constrained colocalization sensitivity. To address this limitation, we therefore performed locus‐of‐interest (LOI) colocalization analyses using chromosome‐specific eQTL results.

**FIGURE 6:**
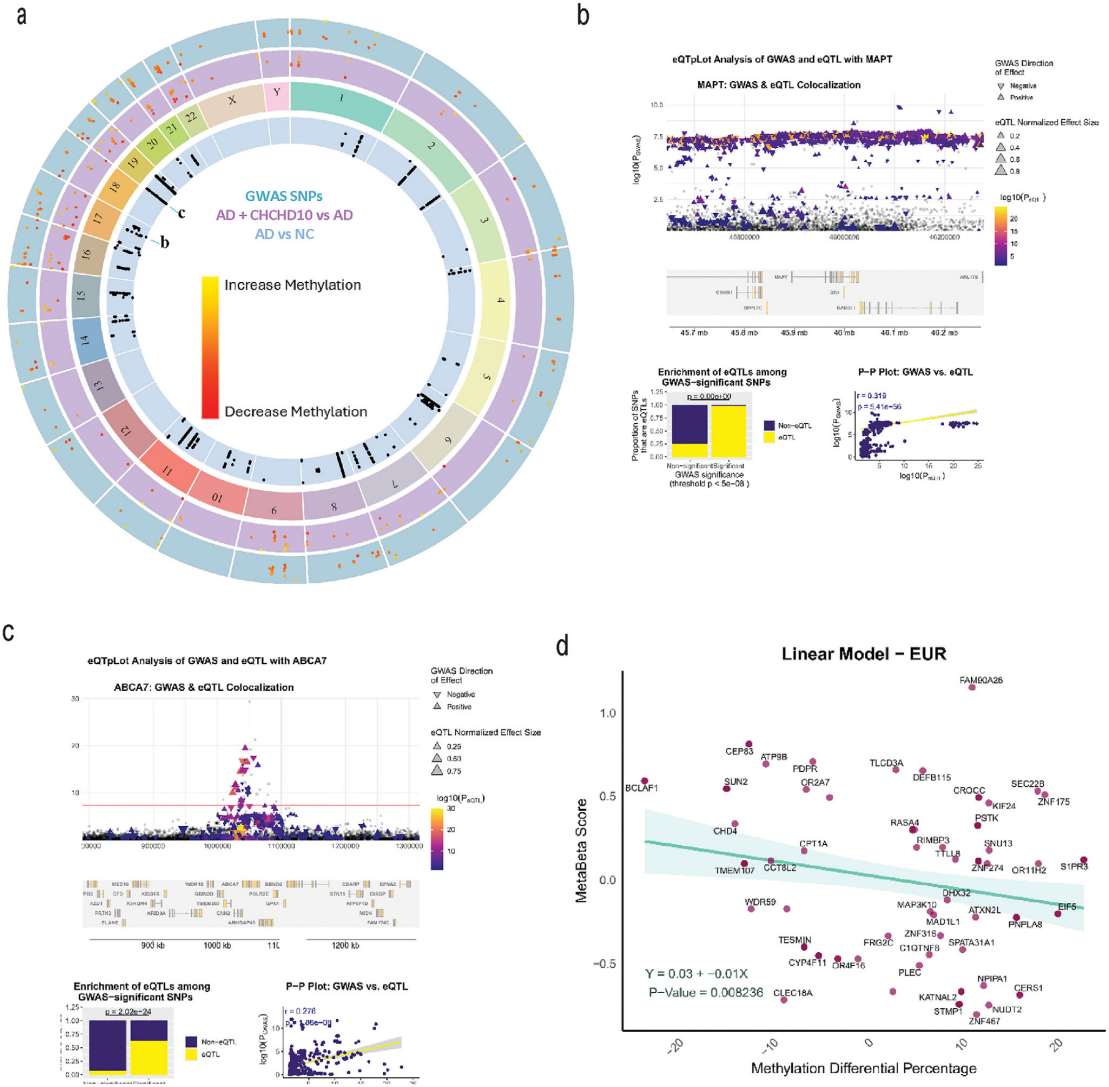
Colocalization of methylation changes and AD‐associated GWAS SNPs. (a) Circos plot of significant DMRs (*p*‐value < 0.05) and GWAS SNPs (*p*‐value = 5 × 10^−8^). (b) MAPT locus eQTpLot: 250 kb window, P–P plot r = 0.318, P‐P plot *p*‐value = 5.46 × 10^−57^. (c) ABCA7 locus eQTpLot: enrichment Fisher's exact test p‐value = 2.02 × 10^−24^; P–P plot r = 0.276, P–P plot p‐value = 1.6 × 10^−8^. (d) Linear model using EUR eQTL data: p‐value = 0.008230.

We next examined the spatial overlap between significant DMRs from both the AD vs. NC and AD + CHCHD10 vs. AD datasets and AD‐associated GWAS SNPs (p < 5 × 10^−^^8^). A circos plot revealed pronounced chromosomal overlap between epigenetic alterations and high‐confidence genetic risk loci (Figure 6a). LOI‐specific analyses were then conducted using MetaBrain chromosome‐level eQTL data across ±275 kb windows. At the MAPT locus, which is central to tau pathology [60, 61], we observed widespread SNP colocalization with strong concordance (P–P plot r = 0.318, p = 5.46 × 10^−^^5^ ^7^; Figure 6b). The ABCA7 locus, a well‐established AD susceptibility gene [62, 63, 64], showed a more localized enrichment centered on the gene, with significant eQTL enrichment among GWAS‐significant SNPs (Fisher's exact p = 2.02 × 10^−^^2^ ^4^) and strong P–P concordance (r = 0.276, p = 1.6 × 10^−^^8^; Figure 6c). Additional AD‐ and neurodegeneration‐associated loci, including TAOK2 [65], TBX6 [66], KANSL1 [67], WNT3 [68], LRRC37A2 [69], LRRC37A [69], ARL17B [70, 71], and GLG1 [72, 73], also demonstrated varying degrees of colocalization (Figure S7a–h).

Finally, to assess the functional relationship between DNA methylation and genetically driven expression changes, we correlated methylation differences with MetaBeta values derived from eQTL analyses. For each gene, the most significant DMR from the AD + CHCHD10 vs. AD comparison was paired with the corresponding eQTL MetaBeta. Linear regression revealed a significant inverse association between methylation and expression in both EUR (p = 0.0082; Figure 6d) and EAS (p = 1.4 × 10^−^^5^; Figure S6b) datasets, whereas no significant relationship was observed in the AFR dataset (p = 0.45; Figure S6a), suggesting population‐specific regulatory effects.

### 2.9 KATNAL2 Is Identified as a Functional Mediator of Tau Pathology

**FIGURE 7:**
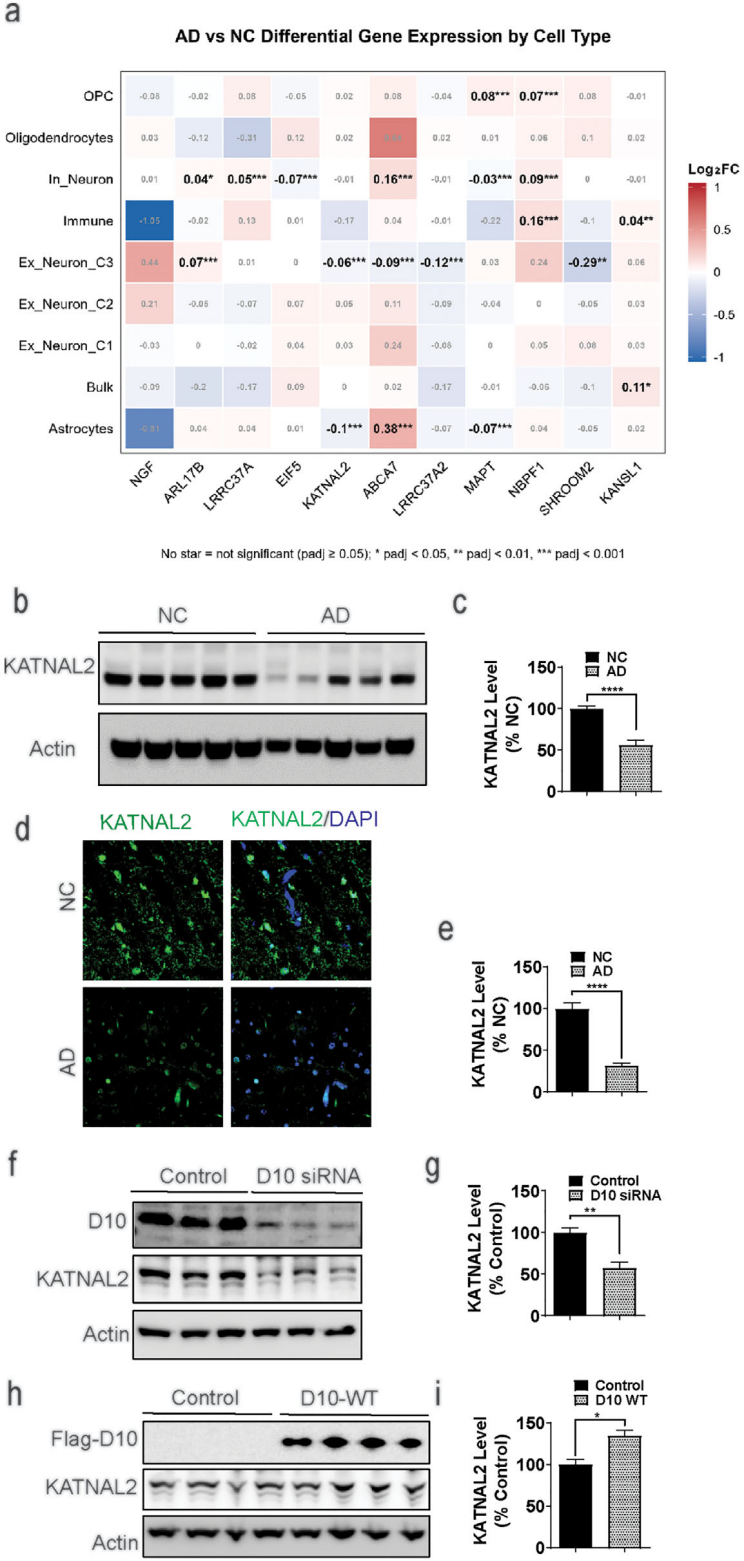
Validation of KATNAL2 expression in AD cortex. (a) scRNA‐seq log2 fold changes of DMR‐associated genes (NC n = 292, AD n = 135). (b) Representative immunoblots of KATNAL2 in human cortex lysate (NC n = 10, AD n = 10). *Note*: Actin loading controls shown here are shared with Figure 2c because CHCHD10 and KATNAL2 were analyzed from the same brain lysates and immunoblot experiment. (c) Quantifications of KATNAL2 protein expression levels from the AD and NC groups. KATNAL2 expression levels from RIPA‐soluble lysate (unpaired *t*‐test ^****^ *p*‐value < 0.0001) (d) Immunofluorescence of KATNAL2 (green) and DAPI (blue) in cortex tissue (NC n = 5, AD n = 5). (e) Quantification of KATNAL2 fluorescence intensity; unpaired *t*‐test ^****^ *p*‐value < 0.0001. (f) Representative immunoblots of KATNAL2 and CHCHD10 from M17‐APP cells with control and CHCHD10 siRNA transfection. (g) Quantifications of KATNAL2 protein expression levels (n = 3, unpaired *t*‐test, ^**^ *p*‐value < 0.01). (h) Representative immunoblots of KATNAL2 and CHCHD10 from M17‐APP cells with control and Flag‐CHCHD10 transfection. (i) Quantifications of KATNAL2 protein expression levels (n = 4, unpaired *t*‐test, ^**^ *p*‐value < 0.05).

Among genes associated with significant DMRs, KATNAL2 emerged as a top candidate because of consistent AD‐associated hypomethylation that was reversed by CHCHD10 overexpression. scRNA‐seq data revealed decreased KATNAL2 expression in cluster 2 excitatory neurons and in astrocytes of AD donors (Figure 7a). Immunoblotting and immunohistochemistry confirmed significantly reduced KATNAL2 protein levels in the AD human cortex (Figure 7b–e). To further evaluate the relationship between CHCHD10 and KATNAL2, we examined KATNAL2 expression following CHCHD10 perturbation in M17‐APP cells. One of the KATNAL2‐immunoreactive bands (slightly above ∼50 kDa) consistently responded to CHCHD10 manipulation, increasing with CHCHD10 overexpression and decreasing with knockdown (Figure 7f–i), supporting a regulatory association between CHCHD10 and KATNAL2. These findings, together with locus‐specific methylation changes at KATNAL2 (Figure 5b and Figure S5a), suggest that CHCHD10 may influence KATNAL2 through coordinated epigenetic and transcriptional mechanisms. Whether CHCHD10‐dependent methylation changes directly mediate KATNAL2 expression requires further investigation.

**FIGURE 8:**
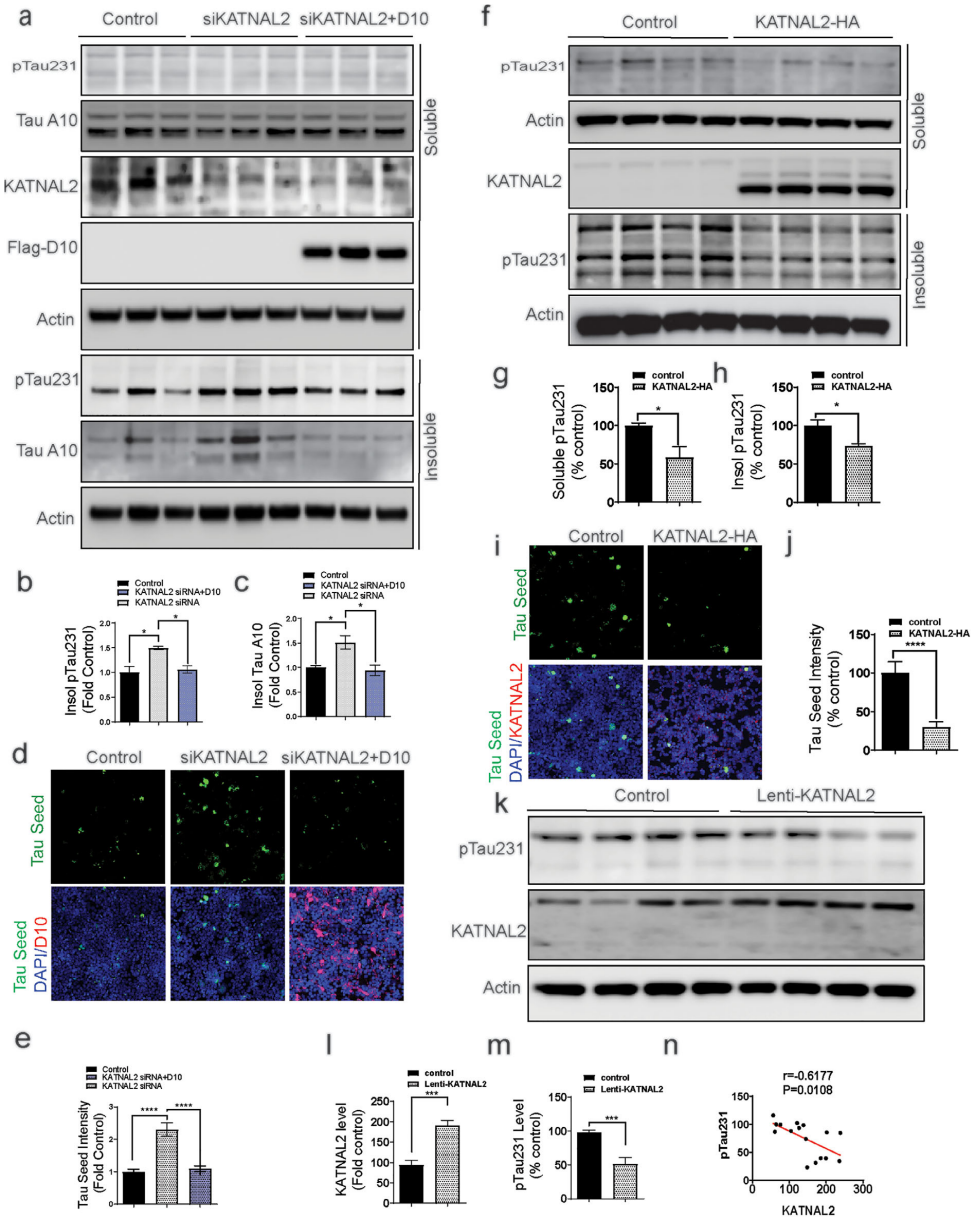
Functional role of KATNAL2 in tau pathology. (a) Immunoblots in HeLa‐V5 cells for control, siKATNAL2, and siKATNAL2+CHCHD10 (n = 3 per group). (b) RIPA‐insoluble pTau231: ANOVA p‐values = 0.017 (Control vs. siKATNAL2), 0.0303 (siKATNAL2 vs. siKATNAL2+CHCHD10). (c) RIPA‐insoluble Tau A10: ANOVA p‐values = 0.0364, 0.0231; unpaired *t*‐test *p*‐values = 0.0218, 0.0305. (d) TauRD seeding experiment representative images (n = 5 per group). (e) Tau seeding fluorescence: ANOVA and *t*‐test *p*‐values < 0.0001 (Control vs. siKATNAL2 and siKATNAL2 vs. siKATNAL2+CHCHD10); no difference between Control and siKATNAL2+CHCHD10 (ANOVA *p*‐value = 0.9502, *t*‐test p‐value = 0.4235). (f) Immunoblots from KATNAL2‐HA OE vs. control (n = 4 per group). (g) RIPA‐soluble pTau231; unpaired *t*‐test *p*‐value = 0.0288. (h) RIPA‐insoluble pTau231; unpaired *t*‐test *p*‐value = 0.0162. (i–j) TauRD seeding assay with KATNAL2‐HA (Control n = 3, KATNAL2‐HA n = 3); *t*‐test *p*‐value < 0.0001. (k–m) Lentiviral KATNAL2 overexpression in AD‐converted neurons (n = 4 per group); KATNAL2 *t*‐test *p*‐value <0.0001, pTau231 *t*‐test *p*‐value = 0.0003. (n) Linear regression: KATNAL2 vs. pTau231 protein levels in Lentiviral KATNAL2 overexpression AD‐converted neurons (r = −0.6117, n = 8, *p*‐value = 0.0108).

KATNAL2 plays a crucial role in neurodevelopment and microtubule dynamics [74, 75, 76, 77]. To examine whether KATNAL2 influences tau pathology, we performed gain‐ and loss‐of‐function experiments. siRNA‐mediated knockdown of KATNAL2 in Tau‐expressing HeLa‐V5 cells increased RIPA‐insoluble pTau231 and total tau (Figure 8b,c), and increased tau seeding activity in TauRD cells (Figure 8d,e). Importantly, CHCHD10 overexpression rescued tau pathology induced by KATNAL2 knockdown, restoring tau phosphorylation and seeding activity to near‐control levels (Figure 8b,e). KATNAL2 overexpression reduced tau phosphorylation and tau seeding in both HeLa‐V5 and TauRD cells (Figure 8f–j). Lentiviral overexpression of KATNAL2 in AD fibroblast‐derived neurons decreased pTau231 levels (Figure 8k–m), and KATNAL2 expression negatively correlated with pTau231 abundance (p = 0.0108) (Figure 8n). In contrast, CHCHD10 knockdown increased insoluble tau, and KATNAL2 overexpression did not rescue CHCHD10 knockdown‐induced tau accumulation (Figure S8a,b), suggesting that KATNAL2 alone is insufficient to fully compensate for CHCHD10 loss. These findings identify KATNAL2 as a CHCHD10‐responsive downstream effector gene whose expression modulates tau pathology, providing a potential mechanistic link between CHCHD10, epigenomic regulation, and tau‐associated neurodegeneration.

## 3 Materials and Methods

### 3.1 Human Neuron Reprogramming and DNA Methylation Sequencing

Four normal control (NC) human fibroblast lines (AG08529, AG08543, AG08525, and AG09181) and four AD fibroblast lines (AG08243, AG06869, AG07375, and AG06264) were obtained from the Coriell Cell Repositories (Camden, NJ, USA). All samples were from Caucasian individuals. Fibroblasts were reprogrammed into neurons following a previously described microRNA‐9/9*‐124–based transdifferentiation protocol [39]. Briefly, primary fibroblasts were transduced with lentivirus containing miRNA‐9/9* (pTight‐9‐124‐BclxL, addgene#60857), phND2‐N174(addgene#31822), phMYT1L‐N174(addgene#66809), and rtTA‐N144 (addgene#66810). Cells were then cultured with doxycycline to induce neuronal gene expression and treated with neural media containing doxycycline, cAMP, valproic acid (VPA), BDNF, NT3, retinoic acid (RA), RVC, and puromycin. Conversion was confirmed by immunoblotting for β3‐tubulin and MAP2. Overexpression of CHCHD10 and KATNAL2 was performed by adenovirus and lentivirus transduction, respectively, at day 21 of differentiation. Cells were harvested on day 28 for analysis.

To generate CHCHD10‐overexpressing neurons, a subset of AD fibroblast‐converted Neurons was transduced with an adenovirus of CHCHD10 on day 21 of differentiation. These samples, alongside untreated NC and AD Neurons, were harvested on day 28 and submitted to Azenta Life Sciences (Cleveland, OH, USA) for whole‐genome bisulfite sequencing (WGBS) and downstream methylome analysis. Library preparation was performed using the NEB Enzymatic Methyl‐seq (EM‐seq) protocol. Briefly, purified genomic DNA was fragmented and processed with the NEBNext Enzymatic Methyl‐seq Kit according to the manufacturer's guidelines. Sequencing libraries were constructed and aligned to the human genome using Bismark v0.23.1. After duplicate reads were removed, methylation calls at individual cytosines were extracted from deduplicated alignments. Differential methylation loci (DML) and differentially methylated regions (DMRs) analyses were conducted using MethylKit. Depending on the duplication status of reads, either Fisher's exact tests or logistic regression models were employed. Resulting data files included significant DMRs (CpG and promoter regions) for two pairwise comparisons: (1) AD vs. NC and (2) AD + CHCHD10 vs. AD (Tables S1–S4).

### 3.2 Human Brain Samples

Post‐mortem human cortical tissue was obtained from the Case Western Reserve University Brain Bank, supported by the Cleveland Alzheimer's Disease Research Center and the NIH NeuroBioBank. Samples were used for both immunohistochemistry and immunoblotting. The NC group included individuals aged 60–92 years with post‐mortem intervals (PMI) ranging from 4 to 25 h. The AD group included individuals aged 67–90 years with PMI of 3 to 26 h. All diagnoses were confirmed by neuropathological evaluation according to NIH consensus criteria. All procedures complied with protocols approved by the Case Western Reserve University Institutional Review Board and bioethics guidelines.

### 3.3 Integration of Genetic (GWAS/eQTL) and Epigenetic (DMR) Analyses

Significant AD‐associated single‐nucleotide polymorphisms (SNPs) were obtained from the Genome‐Wide Association Studies (GWAS) Catalog (https://www.ebi.ac.uk/gwas/), using the dataset reported by Bellenguez et al. [78]. SNPs meeting the genome‐wide significance threshold (*p* < 5 × 10^−^^8^) were considered significant.

Cortex‐specific summary statistics for cis‐expression quantitative trait loci (cis‐eQTLs) were retrieved from the MetaBrain project (https://www.metabrain.nl/cis‐eqtls.html) [79]. Data were collected for European (EUR), African (AFR), and East Asian (EAS) populations. Genome‐wide and locus‐specific colocalization analyses were performed using the top‐effect SNPs and chromosome‐specific cis‐eQTL summary statistics, respectively, to assess the overlap between AD‐associated variants and CHCHD10‐related regulatory regions.

For external validation of differentially methylated regions (DMRs), previously published brain methylation datasets were obtained from the MIAMI‐AD portal (https://miami‐ad.org/). The middle temporal gyrus (MTG) dataset (n = 296; 198 AD and 98 NC) was generated by the Banner Sun Health Research Institute and published in 2023 [80]. The prefrontal cortex (PFC) dataset (n = 708 DMRs), stratified by Braak stage, was obtained from the Religious Orders Study and Memory and Aging Project (ROSMAP) cohort [81, 82].

### 3.4 Bulk RNA‐Seq & Single‐Cell RNA‐Sequencing Analysis

#### 3.4.1 Bulk RNA‐Seq

Human cerebellum and temporal cortex bulk RNA‐seq data (82 AD and 78 NC samples) were obtained from the Mayo Clinic Brain Bank and Banner Sun Health Research Institute via the AD Knowledge Portal developed by Sage Bionetworks [83]. Raw count matrices (*MayoRNAseq_*TCX*_geneCounts.tsv*) and associated metadata were downloaded for analysis in R. Differential expression analysis was performed using the DESeq2 package with the model design ∼ condition (AD vs NC). Low‐abundance genes (fewer than 10 counts in ≥ 20% of samples) were excluded. Log_2_ fold changes were estimated with empirical‐Bayes shrinkage (apeglm), and adjusted *p* values were calculated by the Benjamini–Hochberg method. Genes with |log_2_FC| ≥ 1 and adjusted *p* < 0.05 were considered significant. Gene annotation. Gene identifiers were converted from Ensembl IDs to official gene symbols, gene names, and Entrez gene IDs using org.Hs.eg.db and clusterProfiler.

#### 3.4.2 Single‐Cell RNA‐Seq

Human single‐cell RNA‐seq (scRNA‐seq) data were obtained from the AD Knowledge Portal (project ID syn2580853). Processed scRNA‐seq data by cluster were downloaded under synapse ID syn52293433. Clinical characteristics were linked to unique donor IDs and separated into the AD group if a documented AD onset date existed; donors with no record of AD diagnosis and no neuropathological evidence of AD were classified as NC. Donors lacking either criterion were excluded, resulting in 135 AD and 292 NC individuals included in the scRNA‐seq analyses.

All analyses were performed in Seurat (v4–v5). For datasets not already normalized, expression matrices were processed using *LogNormalize* (scaling factor = 10 000) prior to differential expression analysis. To account for the large size of the datasets, balanced subsampling was applied across all cell types, capping at 6000 cells per group (AD vs NC) and targeting a total of ∼50,000 cells to ensure stable computation and equal group representation.

Cells with fewer than 200 detected genes or more than 5% mitochondrial reads were excluded. Donors contributing fewer than 20 cells were removed to maintain per‐donor stability. Quality‐controlled data were merged with clinical metadata (diagnosis, Braak stage, age at death, *APOE* genotype) by project ID. Differential expression between AD and NC cells was conducted using Seurat's Wilcoxon rank‐sum test (*FindMarkers*) with unfiltered criteria (log_2_FC = 0, min.pct = 0, min.cells.feature = 1), producing unbiased DEG lists for downstream functional analysis. Excitatory neuron subtypes were defined by canonical cluster markers: Cluster 1 (L2‐3 CBLN2 LINC02306 expressing neurons); Cluster 2 (L3‐4 RORB COX2, L3‐5 RORB PLCH1, L4‐5 RORB GABRG1, L4‐5 RORB IL1RAPL2 expressing neurons); and Cluster 3 (L5‐6 RORB LINC02196, L5 ET, L5/6 IT CAR3, L5/6 NP, L6 CT, L6 THEMIS NFIA, L6b, NRGN, and RLN CHD7 expressing neurons) [84].

#### 3.4.3 Per‐Donor Regression (scRNA‐Seq)

For neuron‐focused summaries, per‐donor mean expression values were computed, and simple linear models were fitted relating CHCHD10 expression to Braak stage or to age at death (no covariates), reporting β and *p* as displayed in the figures. Model diagnostics and figure generation were performed in R with ggplot2. All analyses were executed with version‐controlled scripts and logged runtime messages to ensure reproducibility.

### 3.5 Differentially Methylated Region Statistics

To validate the relevance of the methylome data obtained from primary fibroblast‐derived neurons, we assessed whether their DMRs recapitulated methylation patterns observed in human AD brain tissue. External brain‐derived DMR datasets were used for this purpose. The MTG DMR dataset, obtained from the Banner Sun Health Research Institute via the MIAMI‐AD portal, was used for the initial validation. For this dataset, DMR designation was assigned using DNA methylation as a dependent variable and AD/NC designation as the predictor. Adjustments were previously set for age, sex, batch, PMI, cell proportions, and ethnicity [80]. Genes located within 50 kb of each significant DMR (*p*‐value < 0.05) in both the AD vs. NC fibroblast‐derived neuron dataset and the MTG dataset were identified using the TxDb.Hsapiens.UCSC.hg38.knownGene transcript annotation database (https://genome.ucsc.edu/). Overlap between gene sets was quantified using Fisher's exact test, implemented in base R (stats package). The same procedure was applied using the PFC DMR dataset, associated with Braak stage, which was derived from the ROSMAP cohort. This dataset underwent adjustments for neuron proportions, age at death, batch effects, and sex during DMR analysis. For the PFC comparison, a 100 kb gene window was used to allow for more distal regulatory effects.

### 3.6 Quantification of Global Methylation Changes

To assess the overall directionality of methylation alterations across experimental groups, we calculated the average methylation differential percentage among significant DMRs (q‐value < 0.05) in each comparison group (AD vs. NC and AD + CHCHD10 vs. AD). These values were used to quantify the extent of global hypo‐ or hypermethylation. A two‐sample t‐test assuming unequal variances was conducted to determine whether the differences in average methylation differential percentages between groups were statistically significant. A comparison of means test was also performed to provide additional support for observed differences in global methylation levels.

### 3.7 Co‐Methylation Network

A co‐methylation network analysis was conducted to identify structured patterns among DMRs in the AD + CHCHD10 vs. AD aggregate dataset. Only DMRs with p‐values < 0.05 were included in this analysis. All steps were performed in RStudio using the dplyr, igraph, and scales packages. Methylation differential percentages were scaled using the scale () function, yielding values that ranged from −3.62893 to 3.01283. A Euclidean distance matrix was then calculated to assess pairwise distances between scaled methylation profiles. From this distance matrix, a similarity matrix was generated using the transformation:

$$similarity\;matrix=\frac{1}{\left(1+distance\;matrix\right)}$$

Similarity was determined at a threshold of 0.9 to generate an adjacency matrix. The igraph graph_from_adjacency_matrix function utilized this adjacency matrix to generate a network graph with bidirectional edges and no diagonal elements. In addition, the cluster_fast_greedy() function was implemented to ascertain the community within the network, utilizing the igraph package. Vertices with a degree of 0 were removed to create the network graph. Each significant DMR was annotated with either the gene that contains the DMR or, if it is not within a gene, the closest downstream gene. This annotation was employed for labeling and visualization, rather than as part of the co‐methylation network analysis. A final table of each data point in the co‐methylation network results was printed (Table S5).

### 3.8 Pathway Enrichment and Phenotypic Association Analyses

To identify pathways associated with methylation changes, the CpG and promoter DMRs from the AD + CHCHD10 vs. AD dataset were combined into a single aggregate dataset. DMRs with p‐value < 0.05 were retained, and genes within 25 kb of each DMR were retrieved using TxDb.Hsapiens.UCSC.hg38.knownGene. Pathway enrichment analysis was performed using the enrichGO function from the clusterProfiler R package [85], focusing on the GO molecular function database. Gene symbols were converted from Entrez IDs using the bitr function and org.Hs.eg.db. BH‐adjusted p‐values were used to determine statistical significance (Tables S6‐S7). The same procedure was repeated for the AD vs. NC DMR dataset. Overlapping significantly enriched pathways (Wilcoxon test p‐value < 0.05) between the two conditions were identified and recorded (Tables S6‐S7).

For the phenotypic implications of methylation changes, gene set enrichment analysis using the Human Phenotype Ontology (HPO) was conducted via the MetaBrain Network functional enrichment tool (https://network.metabrain.nl/) [79, 86]. Genes within 25 kb of significant (*p*‐value < 0.05) DMRs in both the AD + CHCHD10 vs. AD and AD vs. NC datasets were analyzed separately. Results were downloaded, and significant phenotype terms (Wilcoxon test p‐value < 0.05) were catalogued. Overlapping phenotype associations between the two datasets were noted (Tables S8‐S9).

### 3.9 Epigenetic and Genetic Relationships Assessment

To explore the overlap between epigenetic alterations and genetic risk loci in AD, we first visually identified genomic regions where significant DMRs and AD‐associated GWAS SNPs colocalized. To investigate potential functional convergence at the gene level, we downloaded cortex‐specific cis‐eQTL summary statistics from the MetaBrain Network for European (EUR), African (AFR), and East Asian (EAS) populations [79, 86].

A Bayesian colocalization analysis was then performed between AD GWAS SNPs and the MetaBrain EUR “Top Effects” cis‐eQTL dataset (i.e., top SNP associations per gene with permutation‐based thresholds). The analysis was conducted using the coloc.The abf() function in R. GWAS summary statistics were modeled as case‐control data (dataset 1), and eQTL summary statistics were modeled as quantitative trait data (dataset 2). The analysis produced posterior probabilities for five hypotheses: H0: no causal variant; H1: causal variant for trait 1 only (GWAS); H2: causal variant for trait 2 only (eQTL); H3: two distinct causal variants; H4: a single shared causal variant (Table S10). Following genome‐wide colocalization, locus of interest (LOI) analyses were conducted using chromosome‐specific cortex eQTL data with the eQTpLot R package [87]. To further interpret the relationship between methylation and gene regulation, statistical modeling was applied to associate DMRs from the AD + CHCHD10 vs. AD dataset with MetaBrain “Top Effects” eQTL data. Each DMR was mapped to either the gene in which it was located or, if intergenic, to the nearest downstream gene using the TxDb.Hsapiens.UCSC.hg38.knownGene annotation database (https://genome.ucsc.edu/). When multiple DMRs mapped to the same gene, the DMR with the lowest p‐value was selected.

For each mapped gene, a linear model was used to assess the relationship between MetaBeta (a beta effect score from eQTL data) and the methylation differential percentage. These analyses were conducted separately for each population group (EUR, AFR, EAS). The results of the linear model were visualized (Tables S11–S13).

### 3.10 Ethics Approval

All animal procedures were approved by the Institutional Animal Care and Use Committee (IACUC, Protocol No. 2021‐0033). All experiments were conducted in accordance with institutional guidelines and relevant regulations and were additionally approved by the Institutional Biosafety Committee (IBC, Protocol No. IBC‐2024‐525). Human fibroblast cell lines were obtained from the Coriell Cell Repositories. The use of human brain tissues was reviewed and approved by the Case Western Reserve University Institutional Review Board (IRB, Protocol No. 03‐00‐26) and conducted in accordance with applicable institutional guidelines and regulations.

### 3.11 Mice

APP/PS1;CHCHD10 transgenic mice were generated by crossing APP/PS1 mice [88] with CHCHD10 transgenic mice [89, 90, 91] on a C57BL/6 background. Mice were maintained under standard laboratory conditions with a 12 h light/dark cycle and provided ad libitum access to food and water. The 8‐month‐old APP/PS1; CHCHD10 mice were used for proteomics experiments and PLA assays.

### 3.12 Antibodies

Anti‐amyloid beta (Catalog #8243), anti‐pTau231 (Catalog #71429S), anti‐OGT (Catalog#24083S), and anti‐MAP2 (Catalog #4542S) antibodies were obtained from Cell Signaling (Danvers, MA, USA). Antibodies against β‐actin (Catalog #66009‐1‐Ig) and CHCHD10 (Catalog #25671‐1‐AP) were obtained from Proteintech (Rosemont, IL, USA). Anti‐ZNG1E (CBWD5, Catalog #AP4898c) was purchased from Abcepta (San Diego, CA, USA). The following antibodies were obtained from Invitrogen (Carlsbad, CA, USA): KATNAL2 (Catalog #PA5‐113096) and CHCHD10 (Catalog #MA5‐27535). The anti‐β3‐tubulin antibody (Catalog #SC‐80005) was obtained from Santa Cruz Biotechnology (Dallas, TX, USA).

### 3.13 Cell Culture

HeLa‐V5, HT22, and Tau RD P301S FRET Biosensor (TauRD) cells were cultured in Dulbecco's modified Eagle's medium (DMEM) with 10% fetal bovine serum (FBS) and 1% penicillin‐streptomycin, while human primary fibroblasts were cultured in DMEM with 15% FBS (Catalog #A5670701) (Gibco, Waltham, MA, USA) and 1% penicillin‐streptomycin (Catalog #15140‐122) (Gibco, Waltham, MA, USA). M17‐APP stable cells were cultured in opti‐MEM (Gibco, Waltham, MA, USA) reduced serum medium with 5% FBS and 1% penicillin‐streptomycin.

### 3.14 Generation of M17‐APP Stable Cell Line

Human neuroblastoma M17 cells were transfected with the pCMV6‐AN‐His‐APP‐WT plasmid. Following transfection, cells were reseeded and subjected to selection in Opti‐MEM supplemented with 5% FBS and 500 µg/mL geneticin (G418) for 12 days, with medium refreshed every 96 h. Surviving cells were subsequently maintained in complete medium containing 10% FBS and penicillin/streptomycin. For clonal isolation, cells were seeded at a single‐cell density into 96‐well plates and cultured for approximately 3 weeks. Individual clones were expanded stepwise into larger culture formats. APP overexpression was verified by Western blot, and validated clones were cryopreserved for subsequent experiments.

### 3.15 Tissue Lysis and Immunoblotting

Tissue lysis was performed with RIPA lysis buffer (50 mM Tris, pH 7.4, 150 mM NaCl, 2 mM ethylenediaminetetraacetic acid (EDTA), 1% NP‐40, 0.1% sodium dodecyl sulfate (SDS)). Using a colorimetric detection kit (Quick Start Bradford Protein Assay, Bio‐Rad, Hercules, CA, USA), total protein concentrations were measured. Matched quantities of protein lysates were used for sodium dodecyl sulfate–polyacrylamide gel electrophoresis, then transferred to a 0.2 mm nitrocellulose membrane (Millipore Corporation, Bedford, MA, USA). Primary antibodies were used to analyze proteins of interest, followed by a blocking step performed with 5% skim milk solution. After which, horseradish peroxidase‐conjugated secondary antibodies were applied. Detection was performed via ECL (Merck Millipore Corporation, Darmstadt, Germany). For the CHCHD10 and KATNAL2 immunoblot analyses shown in Figures 2c and 7b, the same human brain tissue lysates and shared Actin loading control were used. Therefore, the same Actin immunoblot is presented in both figures. All immunoblotting images were captured with the LAS‐4000 (GE Healthcare Biosciences, Pittsburgh, PA), then quantified with ImageJ (NIH, Bethesda, MD).

### 3.16 Immunohistochemistry, Immunocytochemistry, and Enzyme‐Linked Immunosorbent Assay (ELISA)

The procedure for the immunohistochemistry analysis for human brain tissues has been previously described [89]. Slices of human cortical tissue were washed with PBS three times, after which antigen retrieval was done with a solution of 10 mM sodium citrate and 0.05% Tween 20 at a pH of 6.0 at 95°C for 5 min. These tissues were then incubated at 4°C overnight in a blocking solution of 0.2% Triton X‐100 and 3% normal goat serum. Then, the tissues were washed 3 times with PBS and stained by TrueBlack from Cell Signal Technologies (Catalog #92401S, Cell Signaling, Boston, MA, USA) to quench autofluorescence, followed by treatment with Alexa‐488, Alexa‐594, and/or DAPI conjugated secondary antibodies (Vector Laboratories, Burlingame, CA) for 1 h at room temperature. Glass slides were washed three times with PBS and mounted using the Vector Laboratories fluorochrome mounting solution. Microscopy imaging was performed using a Nikon AX Ti2 confocal (Tokyo, Japan). Consistent laser power, filter settings, and exposure time were used for all comparative images. Researchers were blinded to conditions during image capture and quantification. Regions of interest were randomly selected, and any brightness or contrast alterations were applied identically to all compared images. For immunocytochemistry (ICC), cells were washed with PBS and then fixed with 4% paraformaldehyde at room temperature. After fixing, the cells were washed with PBS again, blocked at room temperature in blocking solution (0.2% Triton X‐100, 3% normal goat serum in PBS) for an hour, and incubated overnight at 4°C with primary antibody treatment. Then, the cells were washed with PBS, incubated with Alexa‐488, Alexa‐595, and/or DAPI‐conjugated secondary antibodies for another hour at room temperature. The cells were then washed again with PBS before being mounted on glass slides with Flouromount‐G mounting solution (ThermoFisher Scientific, Waltham, MA, USA).

For the ELISA experiment, Aβ42 levels were measured using the human amyloid beta (1‐42) ELISA kit (Catalog#448707, BioLegend, San Diego, CA, USA) according to the protocol provided by the manufacturer. Cell‐cultured medium fibroblast reprogrammed neurons were collected prior to the ELISA assay.

### 3.17 Tau Seeding Activity Assay

Tau seeding experiments were carried out with TauRD cells, which are an engineered HEK293T cell line that stably expresses the P301S Tau repeat domain [92]. These cells were treated with tau seeds collected from the P301S‐Tau mouse brain for 24 h, and tau seeding activity was assessed using immunohistochemistry staining to determine direct fluorescence intensity. Tau seed preparation has been described previously [93]. Five 8‐month‐old P301‐Tau mouse brains were mixed and homogenized (10% weight/volume) in DPBS and centrifuged at 500 g for 5 min. The supernatant was centrifuged at 1000 g for 5 min before being tested for protein content. The tau seeds were sonicated before the treatment of TauRD cells.

### 3.18 DNA Plasmid and siRNA Transfection

The pcDNA3.2‐KATNAL2 construct was purchased from Genscript Biotech (Catalog #U2060PPWG0‐3/UPA70081) (Piscataway, NJ, USA), siKATNAL2 from Santa Cruz Biotechnology (Catalog #sc‐75365) (Dallas, TX, USA), and the Lenti‐KATNAL2 virus was from Abmgood (Catalog #V25H06K) (Richmond, BC, Canada). The siRNA of human CHCHD10 (5′‐UGAAGCAGUGCAAGUACUA‐3′) was synthesized by GE Dharmacon or Horizon Discovery (Lafayette, CO, USA). The DNA plasmid transfections were performed using Fugene HD (Promega, Madison, WI, USA) in Opti‐MEM (Invitrogen, Carlsbad, CA, USA) according to the manufacturer's instructions. DNA plasmid transfections were harvested 48 h post‐transfection. The siRNA transfections were performed using Lipofectamine 2000 (Invitrogen, Carlsbad, CA, USA) in Opti‐MEM according to the manufacturer's instructions. The siRNA transfections were harvested 72 h post‐transfection for analysis.

### 3.19 Proximity Ligation Assay (PLA)

Proximity ligation assays (PLA) were performed using the Duolink in situ PLA kit (Sigma‐Aldrich) according to the manufacturer's instructions. Fixed cells or tissues were permeabilized and blocked in blocking buffer containing 0.2% Triton X‐100 and 3% normal goat serum for 1 h at room temperature. Samples were then incubated overnight at 4°C with primary antibodies diluted in blocking buffer. After washing with PBS, samples were incubated with oligonucleotide‐conjugated PLA probes (PLUS and MINUS; Sigma–Aldrich) for 30 min at 37°C. Ligation and rolling circle amplification were performed using the Duolink reagents according to the manufacturer's protocol. Following amplification, samples were counterstained and mounted using mounting medium with DAPI for imaging.

### 3.20 Immunoprecipitation and Proteomics

Cortical brain tissues from four 8‐month‐old female APP/PS1;CHCHD10 mice were homogenized in RIPA buffer. Lysates were subjected to immunoprecipitation (IP) using anti‐Flag antibody and Protein A agarose beads. One sample was processed with Protein A agarose beads alone (no antibody) as a negative control. Immunoprecipitated proteins were resolved by SDS‐PAGE, and gel bands were excised for in‐gel digestion. Gel slices were washed with water, dehydrated in acetonitrile, reduced with dithiothreitol (DTT), and alkylated with iodoacetamide. Proteins were digested in‐gel using trypsin (10 ng/µL in 50 mM ammonium bicarbonate) overnight at room temperature. Peptides were extracted twice with 50% acetonitrile/5% formic acid, combined, and dried in a SpeedVac. Samples were resuspended in 1% acetic acid prior to LC–MS analysis. Peptide samples were analyzed using a Bruker timsTOF Pro2 Q‐TOF mass spectrometer coupled to a CaptiveSpray ion source (Bruker Daltonik). Peptides were separated on a 15 cm × 75 µm inner diameter C18 reversed‐phase column (ReproSil AQ, 1.9 µm, 120 Å) at a flow rate of 0.3 µL/min using an acetonitrile/0.1% formic acid gradient. Data were acquired in positive ion mode using Parallel Accumulation–Serial Fragmentation (PASEF) in data‐dependent acquisition (DDA) mode. TIMS‐MS survey scans were acquired over a range of 100–1700 m/z and 0.60–1.6 Vs/cm^2^ with a ramp time of 166 ms. Each cycle consisted of one TIMS‐MS scan followed by 10 PASEF MS/MS scans, with a total cycle time of 1.2 s. Precursor ions with charge states of 2–5 were selected with an intensity threshold of 2500 a.u. and a target value of 20 000 a.u. and dynamically excluded for 0.4 s. Collision energies ranged from 20 to 59 eV, depending on ion mobility.

For proteomics data analysis, mass spectrometry output from the MaxLFQ table was imported and processed in R. The experimental design included one bead‐only control sample and three CHCHD10 IP replicates. Protein detection was first assessed based on non‐zero LFQ intensity values. A protein was considered present in the CHCHD10 IP condition if it was detected in at least 2 of 3 IP replicates. For each protein, average LFQ intensity across CHCHD10 IP samples and replicate‐level IP‐to‐control ratios were calculated. Candidate interactors were classified into multiple confidence tiers. Strong candidates were defined as proteins detected in ≥2 of 3 CHCHD10 IP replicates and absent in the bead‐only control. High‐confidence candidates were further defined as those with spectral counts ≥10 in all three CHCHD10 IP replicates. For downstream ranking, log2 enrichment was calculated as the ratio of the average CHCHD10 IP LFQ intensity to the control LFQ intensity. Proteins were ranked based on enrichment for visualization and downstream interpretation.

### 3.21 Statistical Analysis

Statistical analyses of all graphs were performed using GraphPad Prism 8.0 (GraphPad Software, San Diego, CA, USA) and R software. Data are presented as mean ± SEM unless otherwise indicated. Comparisons between two groups were performed using unpaired Student's *t*‐tests, whereas comparisons among multiple groups were performed using one‐way ANOVA followed by appropriate post hoc tests as indicated in the figure legends. Differential gene expression and DNA methylation analyses were performed using the statistical methods implemented in DESeq2 and MethylKit. Correlation and linear regression analyses were performed where appropriate. Statistical significance was defined as *p* < 0.05.

## 4 Discussion

Mitochondrial dysfunction, disrupted cellular stress responses, and widespread epigenomic alterations are increasingly recognized as convergent drivers of neuronal vulnerability in AD [33, 94]. However, the molecular mechanisms linking mitochondrial stress to chromatin dysregulation remain poorly understood. In this study, we identify CHCHD10, a mitochondrial intermembrane space protein previously implicated in FTD, ALS, and mitochondrial myopathy, as a previously unrecognized modulator of the neuronal epigenome in AD. Our findings demonstrate that CHCHD10 expression is significantly reduced in AD across multiple independent human datasets, and that restoration of CHCHD10 in human AD fibroblast‐reprogrammed neurons mitigates both amyloid and tau pathology while reversing widespread AD‐associated DNA methylation abnormalities in vitro cell models. Furthermore, we identify KATNAL2 as a CHCHD10‐responsive effector that functionally modulates tau phosphorylation and seeding, establishing a mechanistic association between CHCHD10, epigenomic remodeling, and tau‐driven neurodegeneration. This is further shown by the ENCODE4 Registry of cCREs showing multiple elements neighboring the methylation reversals within the KATNAL2 gene. These findings support a correlation and collectively strengthen the evidence connecting CHCHD10‐associated methylation changes with AD pathology; however, further studies are needed to elucidate the underlying molecular mechanisms. While CHCHD10 restoration is associated with both epigenomic remodeling and phenotypic improvement, our data do not establish whether DNA methylation changes are necessary or sufficient drivers of these effects.

Our analyses across scRNA‐seq, bulk RNA‐seq, and proteomic datasets consistently revealed reduced CHCHD10 expression in AD cortex, with the strongest transcriptomic reductions observed in L5–L6 RORB‐positive excitatory neurons and inhibitory neurons [84]. Both neuronal populations are among the earliest and most selectively vulnerable cell types in AD [95, 96]. Indeed, CHCHD10 expression negatively correlated with Braak stage in both excitatory and inhibitory neurons, suggesting that CHCHD10 decline accompanies or precipitates tau pathology progression. The age‐associated decline in CHCHD10 expression observed in human neurons additionally supports the possibility that reduced CHCHD10 expression contributes to age‐dependent cellular vulnerability. Collectively, these findings establish CHCHD10 loss as a reproducible molecular hallmark of AD.

Restoration of CHCHD10 in AD neurons significantly reduced Aβ production and insoluble tau accumulation, implying broad neuroprotective effects. Given CHCHD10's role in maintaining cristae structure, respiratory chain function, and mitochondrial DNA [97, 98, 99], its loss may exacerbate mitochondrial stress and impair the mitochondria‐nucleus communication required for chromatin maintenance. Prior work has shown that mitochondrial dysfunction reduces methyl donor availability [100], alters TET enzyme activity [101], and disrupts metabolic pathways governing DNA methylation [102] and histone modification [103]. Thus, CHCHD10 decline may propagate mitochondrial stress signals that lead to epigenomic destabilization. Our findings that CHCHD10 overexpression reverses AD‐associated hypomethylation and restores methylation in CpG islands and promoters support this model. It remains to be determined whether CHCHD10 directly regulates these changes or instead represents an important contributor within the broader pathological context. Importantly, AD fibroblast‐reprogrammed neurons recapitulated methylation dysregulation observed in postmortem cortex with various genomic window sizes, validating the relevance of our epigenomic analyses in this model system.

In addition to these mitochondrial‐driven mechanisms, our data suggest a more direct association between CHCHD10 and nuclear epigenetic regulation. We observed partial nuclear localization of CHCHD10 in both neuronal models and human AD neurons, supporting the possibility of involvement in chromatin‐associated processes. Furthermore, proteomic analysis of CHCHD10 immunoprecipitates from APP/PS1;CHCHD10 mouse cortical brains identified an interaction with OGT, a key regulator of transcriptional and epigenetic pathways. This interaction was further supported by proximity‐based assays, indicating a close association between CHCHD10 and OGT in situ. Given the established role of OGT in chromatin regulation and its functional interplay with epigenetic modifiers [48, 104, 105], these findings provide a plausible association between mitochondrial CHCHD10 function and the DNA methylation alterations observed in AD. While further studies are required to define causal relationships, our results support a model in which CHCHD10 may be associated with OGT‐linked epigenetic regulatory pathways. However, these findings do not establish direct mechanistic control of DNA methylation between CHCHD10 and OGT.

CHCHD10‐responsive DMRs were enriched for molecular pathways implicated in AD, including ATPase activity, cysteine‐type peptidase activity, and ubiquitin‐dependent processes. These pathways align with known deficits in mitochondrial energetics, proteostasis, and synaptic maintenance observed in AD [106, 107]. Phenotype enrichment analyses further linked CHCHD10‐responsive methylation to human phenotypes associated with motor neuron dysfunction, gliosis, and inflammatory responses, suggesting that CHCHD10 may regulate epigenetic programs with broad involvement in neurodegeneration.

Our co‐methylation network analysis revealed eight distinct DMR communities, with hub loci mapping to genes involved in RNA processing and microtubule dynamics. Interestingly, the spliceosomal protein SNU13 emerged as a key node, consistent with mounting evidence that alternative splicing and spliceosomal dysfunction contribute to tauopathies and AD [54, 108]. These findings raise the possibility that CHCHD10 stabilizes chromatin at regulatory elements governing RNA splicing and cytoskeletal maintenance.

Integration of CHCHD10‐responsive methylation with AD genetic risk architecture revealed notable overlap with AD GWAS loci, including MAPT, ABCA7, and KANSL1. Locus‐of‐interest colocalization analyses demonstrated significant SNP concordance at these sites, particularly in the MAPT region, where tau pathology is most strongly linked to AD progression. The convergence of mitochondrial, epigenomic, and genetic risk signals at the MAPT locus highlights a potential mechanism by which mitochondrial dysfunction may exacerbate susceptibility encoded by inherited risk variants. Furthermore, significant negative correlations between DMR methylation and eQTL effect sizes in EUR and EAS populations suggest that CHCHD10 may exert functional influence on gene expression through methylation modulation.

Among DMR‐associated genes, KATNAL2 emerged as a compelling CHCHD10‐responsive effector. KATNAL2 contains multiple intronic DMRs that exhibited hypomethylation in AD and hypermethylation following CHCHD10 restoration. Notably, these DMRs are localized to annotated regulatory regions, including sites proximal to CTCF‐associated elements, suggesting potential involvement of higher‐order chromatin organization. Expression analyses in scRNA‐seq and human cortex confirmed reduced KATNAL2 expression in AD. KATNAL2 encodes a microtubule‐severing protein implicated in cytoskeletal dynamics, neurodevelopment, and autism spectrum disorder phenotypes [74, 109]. Although its role in tau pathology has not previously been defined, microtubule‐severing proteins can directly influence tau aggregation, axonal structure, and cytoskeletal stability [110]. Our functional assays demonstrate that KATNAL2 knockdown increases tau phosphorylation and tau seeding activity, while KATNAL2 overexpression reduces these pathogenic phenotypes. Importantly, CHCHD10 overexpression rescues tau pathology induced by KATNAL2 loss, supporting KATNAL2 as a downstream effector within a CHCHD10‐associated neuroprotective pathway. While KATNAL2 overexpression mitigated tau pathology, it did not rescue CHCHD10 knockdown–induced tau accumulation, suggesting that CHCHD10‐mediated epigenomic regulation engages multiple downstream effectors beyond KATNAL2. These findings identify KATNAL2 as one of the key responsive effectors that potentially contribute to the regulation of tau dynamics, linking mitochondrial function, DNA methylation, and cytoskeletal integrity.

An important consideration is whether the epigenetic alterations observed in AD neurons represent causal drivers of pathology, terminal consequences, or adaptive responses to disease‐associated stress. Although our data do not establish direct causality, the partial reversal of AD‐associated methylation patterns following CHCHD10 restoration—together with reduced Aβ and tau pathology—suggests that these changes are not fixed end‐stage marks but instead reflect a dynamic and potentially reversible cellular state. However, given the use of directly reprogrammed neurons and the lack of temporal resolution, we cannot determine the sequence of these events, and the observed epigenomic alterations may represent a combination of compensatory and pathological processes.

Although our study identifies CHCHD10 as a potential modulator of DNA methylation and tau‐related pathology, several limitations remain. First, while direct‐reprogrammed neurons preserve disease‐relevant methylation patterns, they do not fully replicate the in vivo neuronal microenvironment, including glial interactions, extracellular matrix cues, and long‐range circuitry. These reprogrammed neurons also have the possibility of retaining residual features of fibroblasts, despite our validation of the DMR regions with previously established brain data. The reprogramming process itself can introduce or erase DNA methylation marks. Therefore, this system does not allow definitive inference of causal relationships between epigenomic changes and AD pathology. Second, we have not yet established whether CHCHD10 overexpression in vivo can restore methylation or ameliorate AD phenotypes in animal models. Third, although our methylome analyses demonstrate broad reversal of AD‐associated hypomethylation, the molecular machinery through which CHCHD10 influences DNA methylation remains unknown. Potential mechanisms may involve modulation of mitochondrial metabolites required for methyl donor generation, effects on RNA granules that regulate nuclear–mitochondrial communication, mitochondrial and metabolic stress relations, or interactions with DNA methyltransferases and TET enzymes. Future studies examining these potential mechanisms will be essential to delineate the causal pathways underlying CHCHD10's epigenomic effects. Fourth, we note that the assignment of DMRs to genes is sensitive to parameter choices, including window size, and that no standardized approach currently exists. Accordingly, our analyses should be interpreted as identifying putative associations rather than direct regulatory relationships. In addition, population‐specific differences in methylation–eQTL coupling suggest that genetic background may modulate CHCHD10‐mediated epigenomic responses. The absence of methylation–eQTL correlation in AFR datasets highlights the need for broader inclusion of diverse genetic ancestries in epigenomic studies of AD.

Despite these limitations, our findings support CHCHD10 as a previously unrecognized neuroprotective factor that partially reverses AD‐associated epigenomic alterations and attenuates pathogenic tau phenotypes. By identifying KATNAL2 as a functionally validated CHCHD10‐responsive gene, we reveal a CHCHD10‐associated network linking mitochondrial dysfunction, DNA methylation, and tau pathology. These results provide a foundational framework for exploring CHCHD10 as a therapeutic target and suggest that augmenting CHCHD10 function may represent a strategy to restore chromatin stability, reduce protein aggregation, and slow neurodegeneration in AD.

## Conflicts of Interest

The authors declare no conflict of interest.

## Supporting information




**Supporting File 1**: advs76205‐sup‐0001‐SuppMat.pdf.

**Supporting File 2**: advs76205‐sup‐0002‐SupplementalFigures.pdf.

**Supporting File 3**: advs76205‐sup‐0003‐Supplemental_Tables_S1‐S13.xlsx.

**Supporting File 4**: advs76205‐sup‐0004‐data.zip.

## Data Availability

Publicly available datasets used in this study include human postmortem brain single‐cell RNA‐seq data from the ROSMAP Synapse repository from the AD Knowledge Portal (syn2580853). Processed scRNA‐seq data by cluster were downloaded under synapse ID syn52293433. Bulk RNA‐seq data from the ROSMAP cohort (Synapse ID: syn8456629), and cortex‐specific eQTL data from the MetaBrain consortium (https://www.metabrain.nl/cis‐eqtls.html). AD GWAS summary statistics were obtained from the GWAS Catalog (GCST90027158). All custom scripts used for methylation processing, genomic windowing, GWAS–eQTL integration, and statistical analyses are available from the corresponding author upon reasonable request. Processed DNA methylation data, including normalized methylation values, count matrices, and associated sample metadata, are provided in the Supplementary Materials and deposited in GEO under accession GSE328184. The code used for analysis and figure generation is available at GitHub: https://github.com/tliu203/CHCHD10‐Alleviates‐Alzheimer‐s‐Disease‐Pathogenesis‐by‐Modulating‐Epigenetic‐Landscape‐.git. Raw data are partially available due to an unrecoverable data storage error that occurred after the processed datasets were generated.
