# Supplementary material for: CHCHD10 Mitigates Alzheimer's Disease‐Related Phenotypes in Association With Epigenetic Remodeling in Directly Reprogrammed Neurons
Source: Adv Sci (Weinh). 2026 Jun 25;13(51):e76205. doi: 10.1002/advs.76205 (PMC13337126; doi:10.1002/advs.76205)

Supplemental Fig. 1

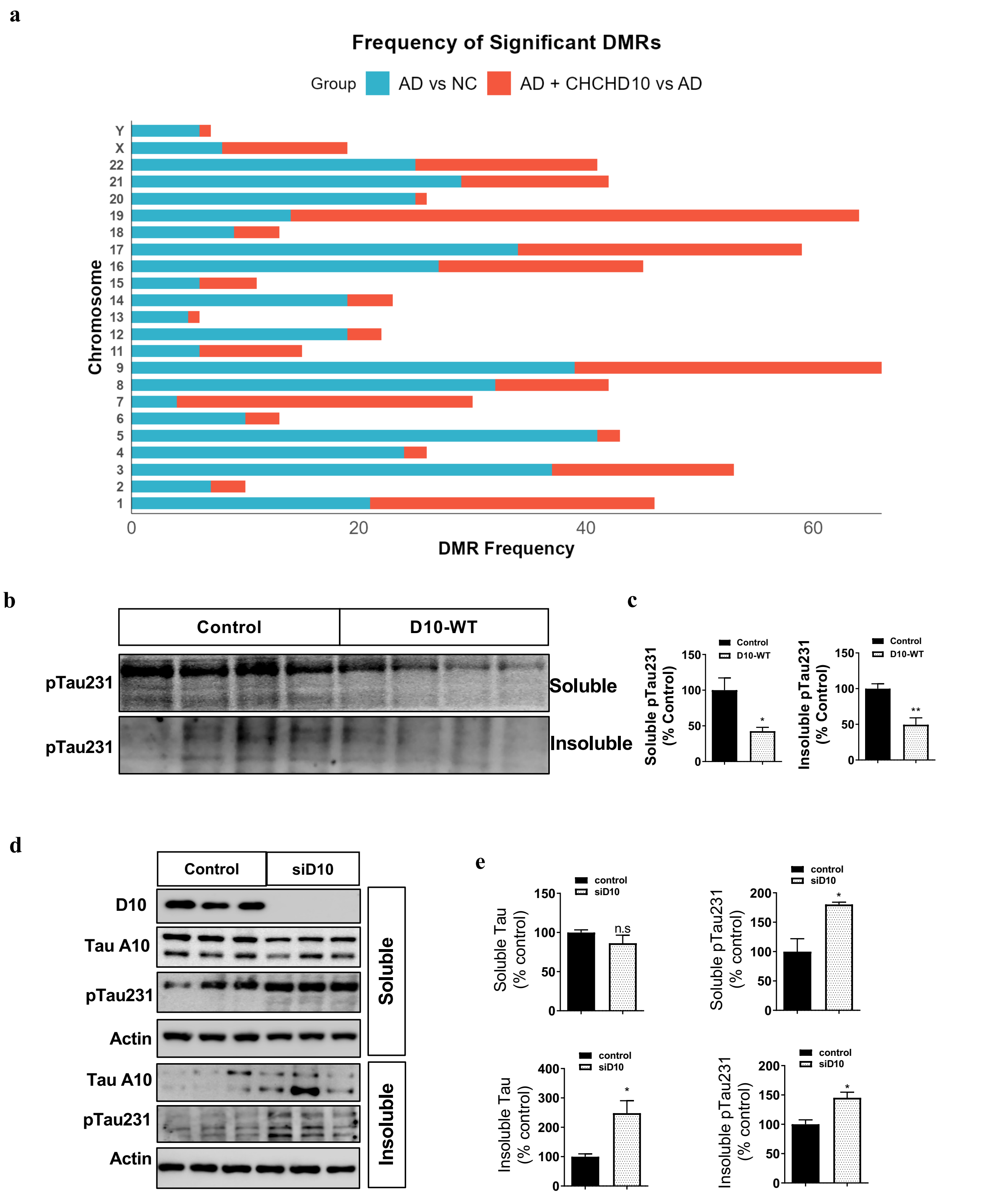

Supplemental Fig.2

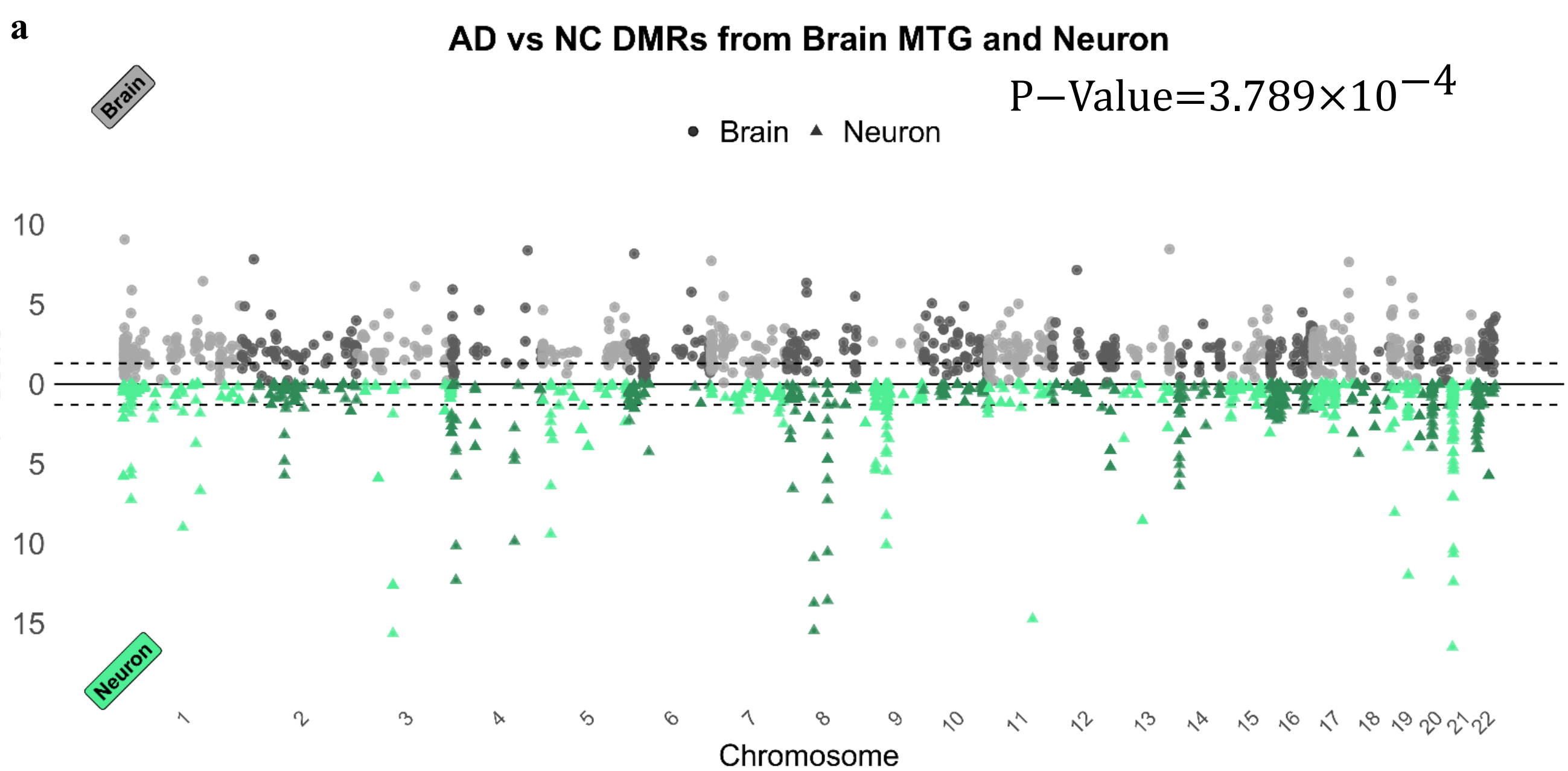

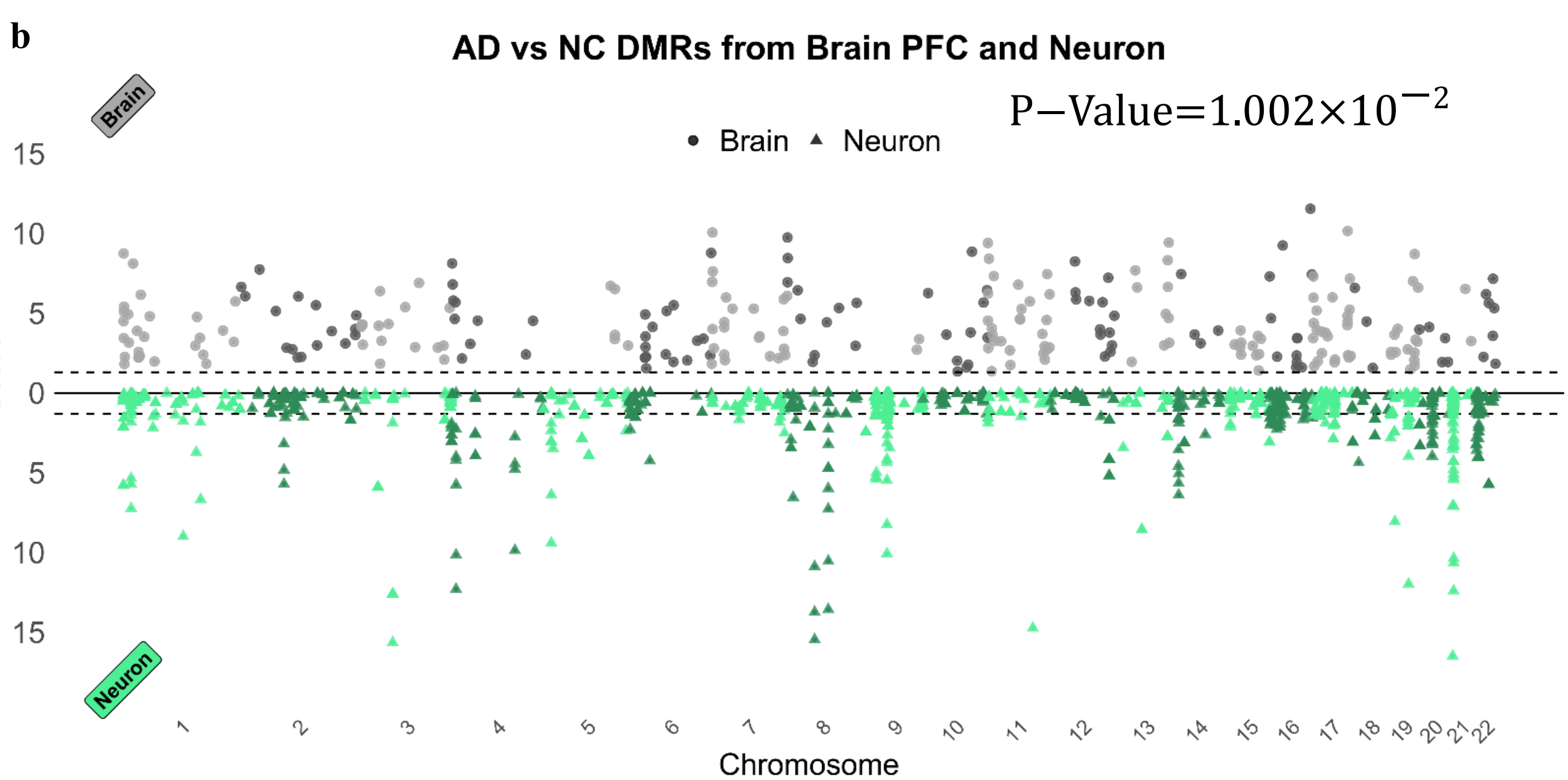

Supplemental Fig.3

a

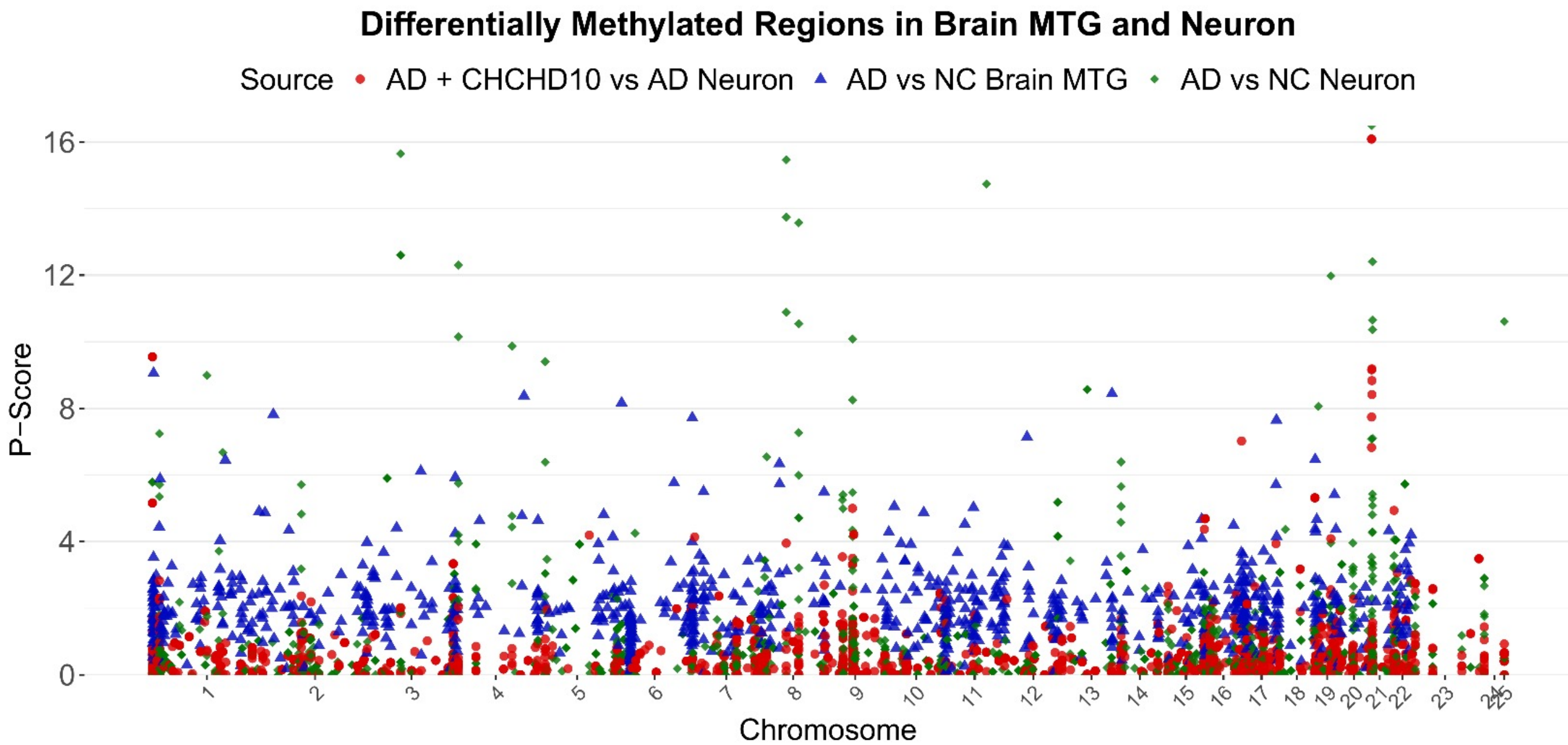

b

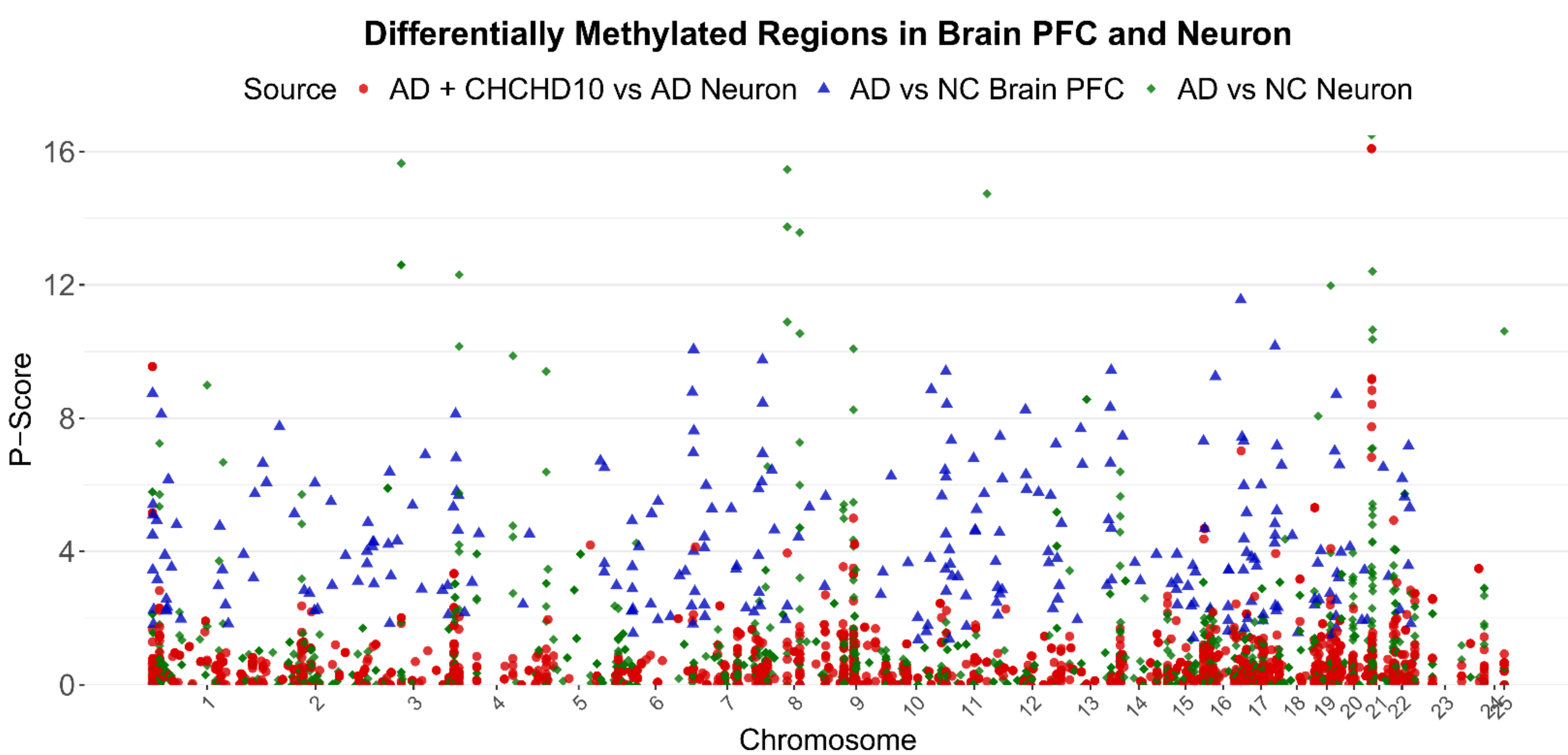

Supplemental Fig. 4

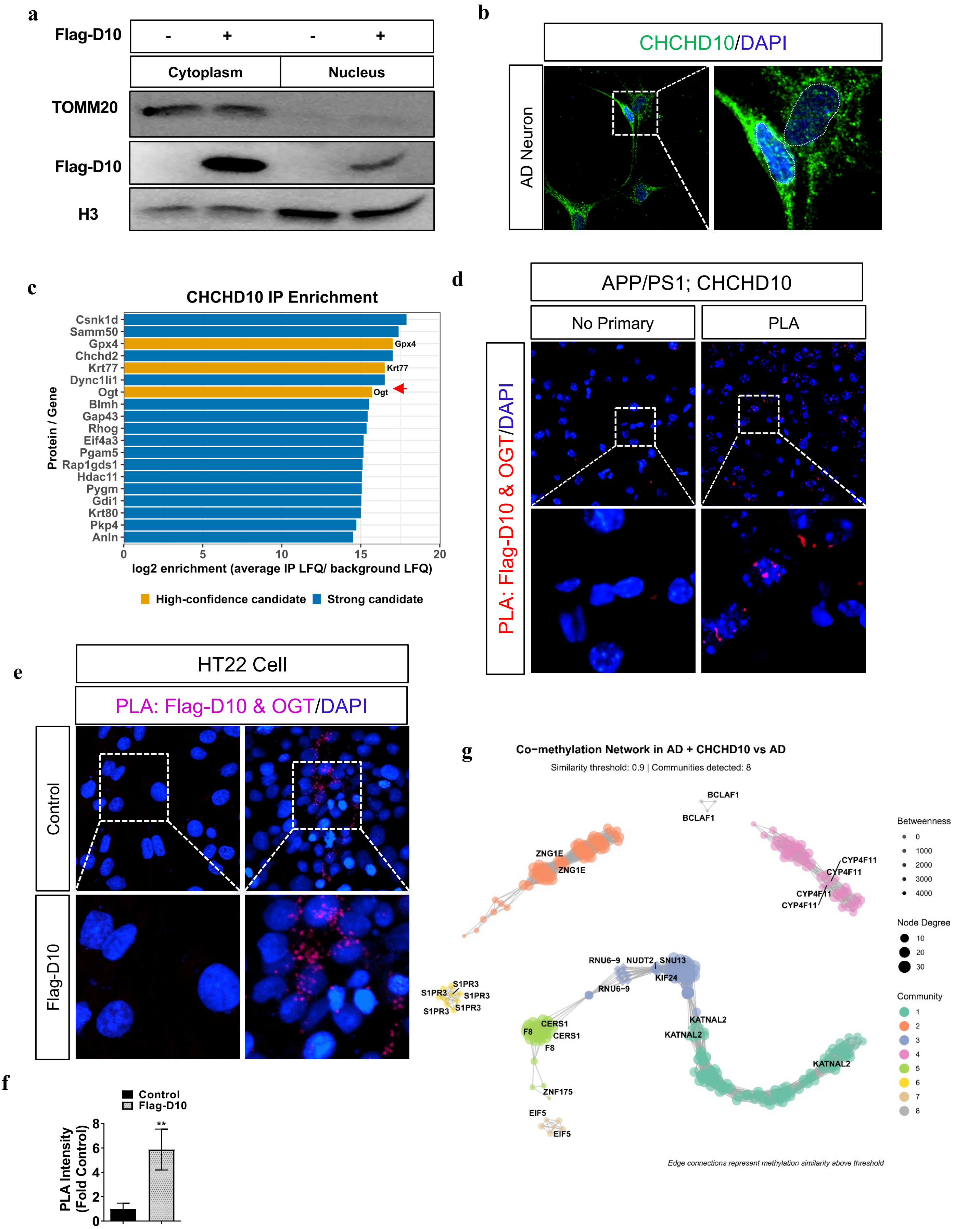

Supplemental Fig. 5

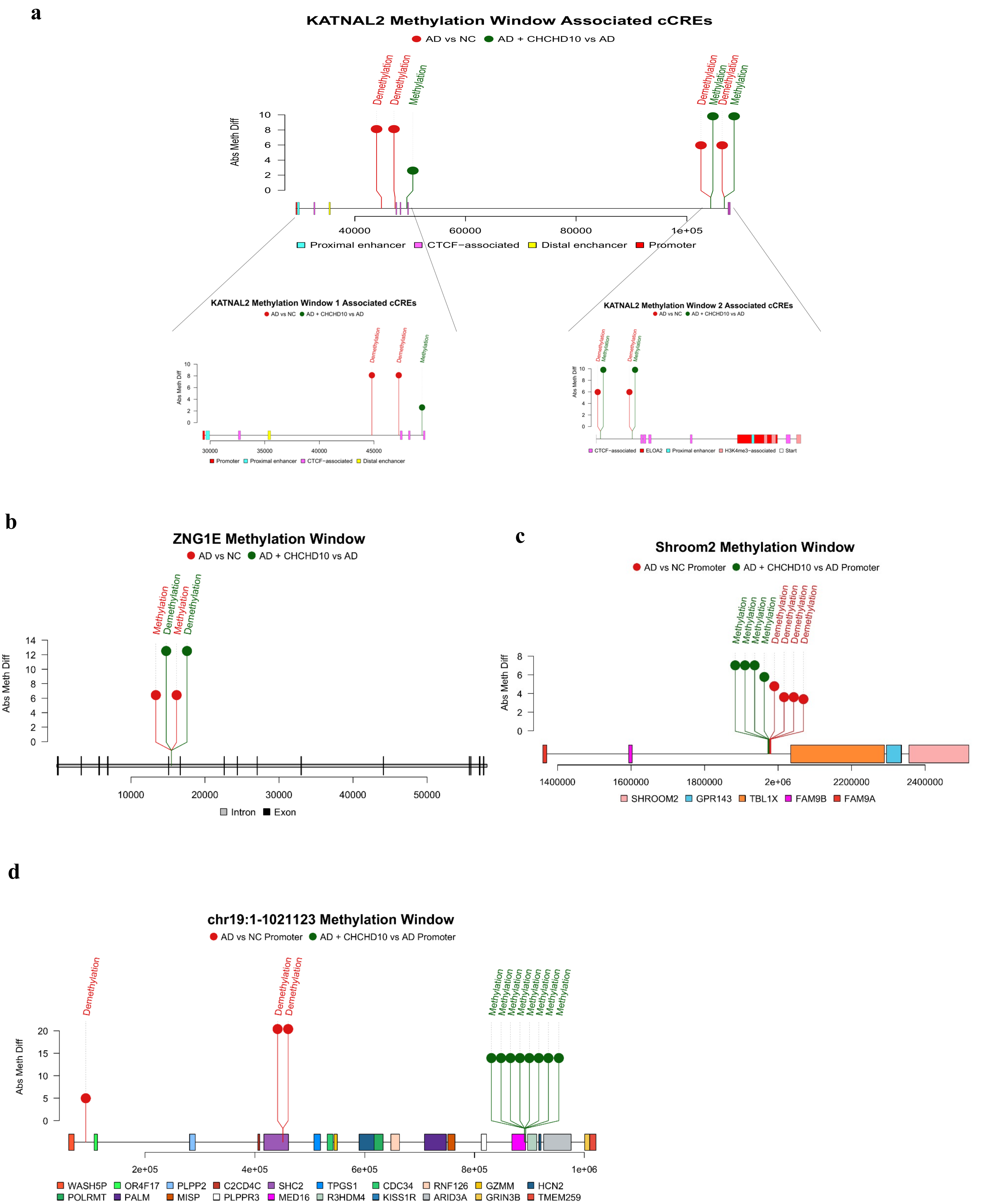

Supplemental Fig. 6

a

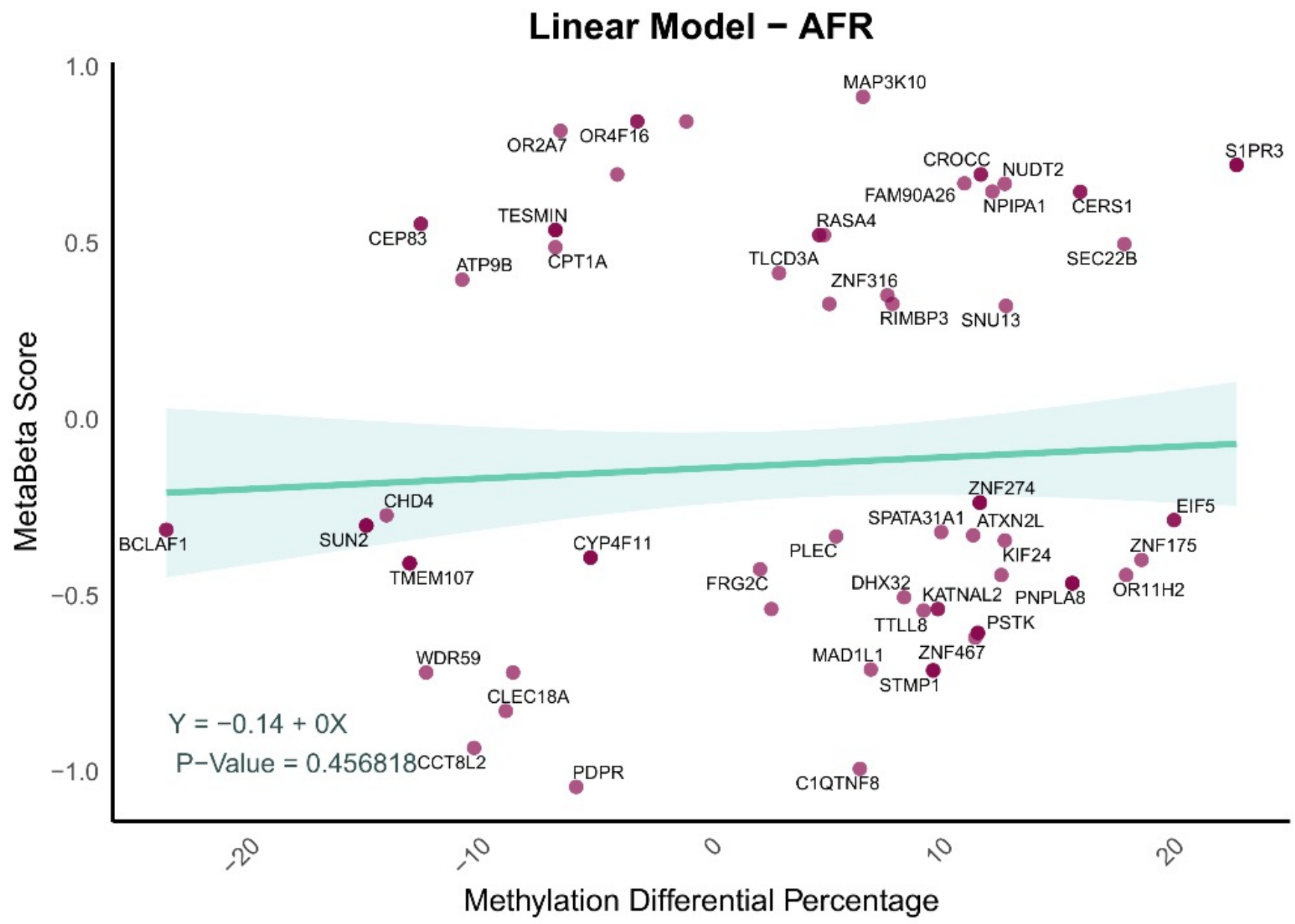

b

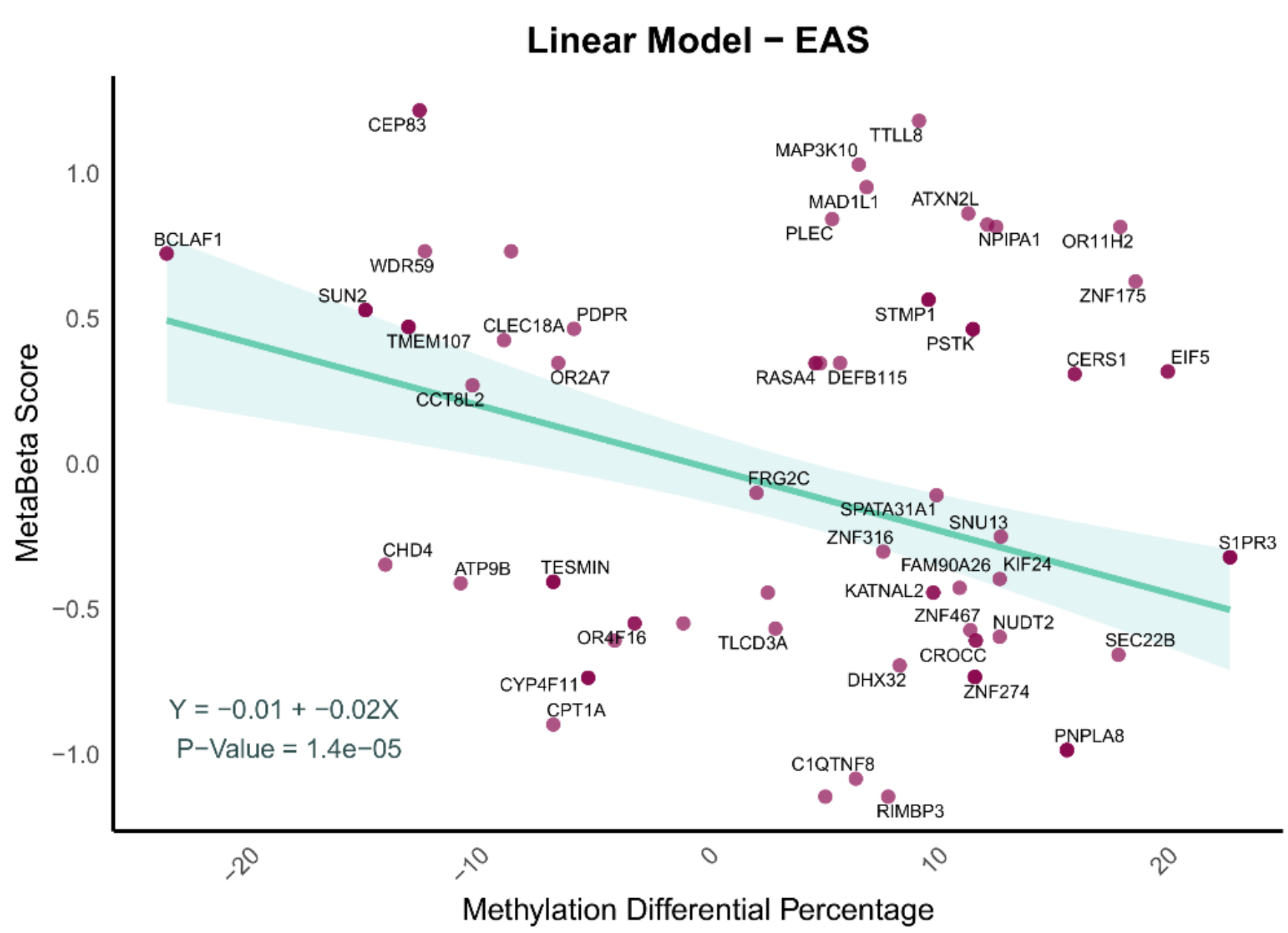

Supplemental Fig. 7

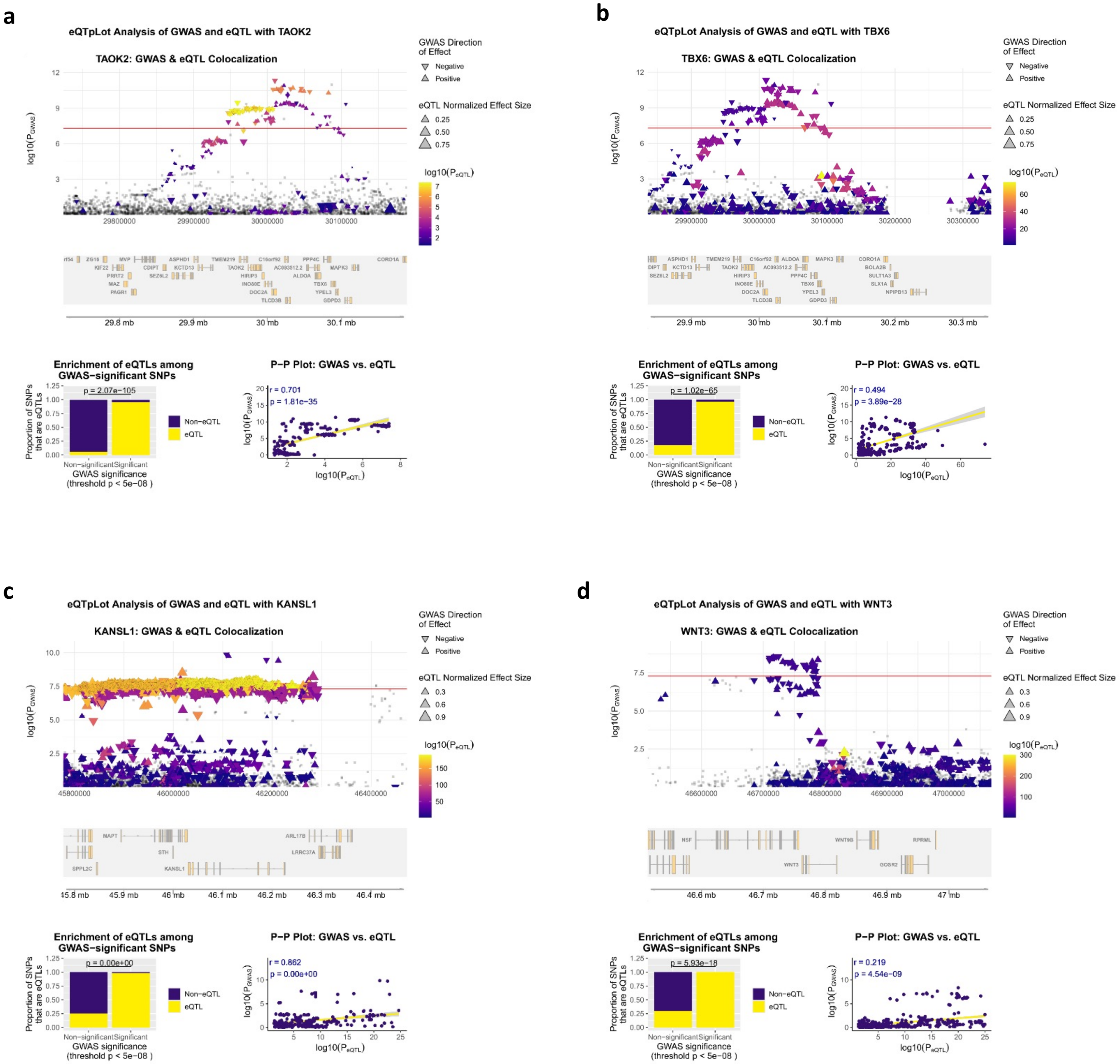

e

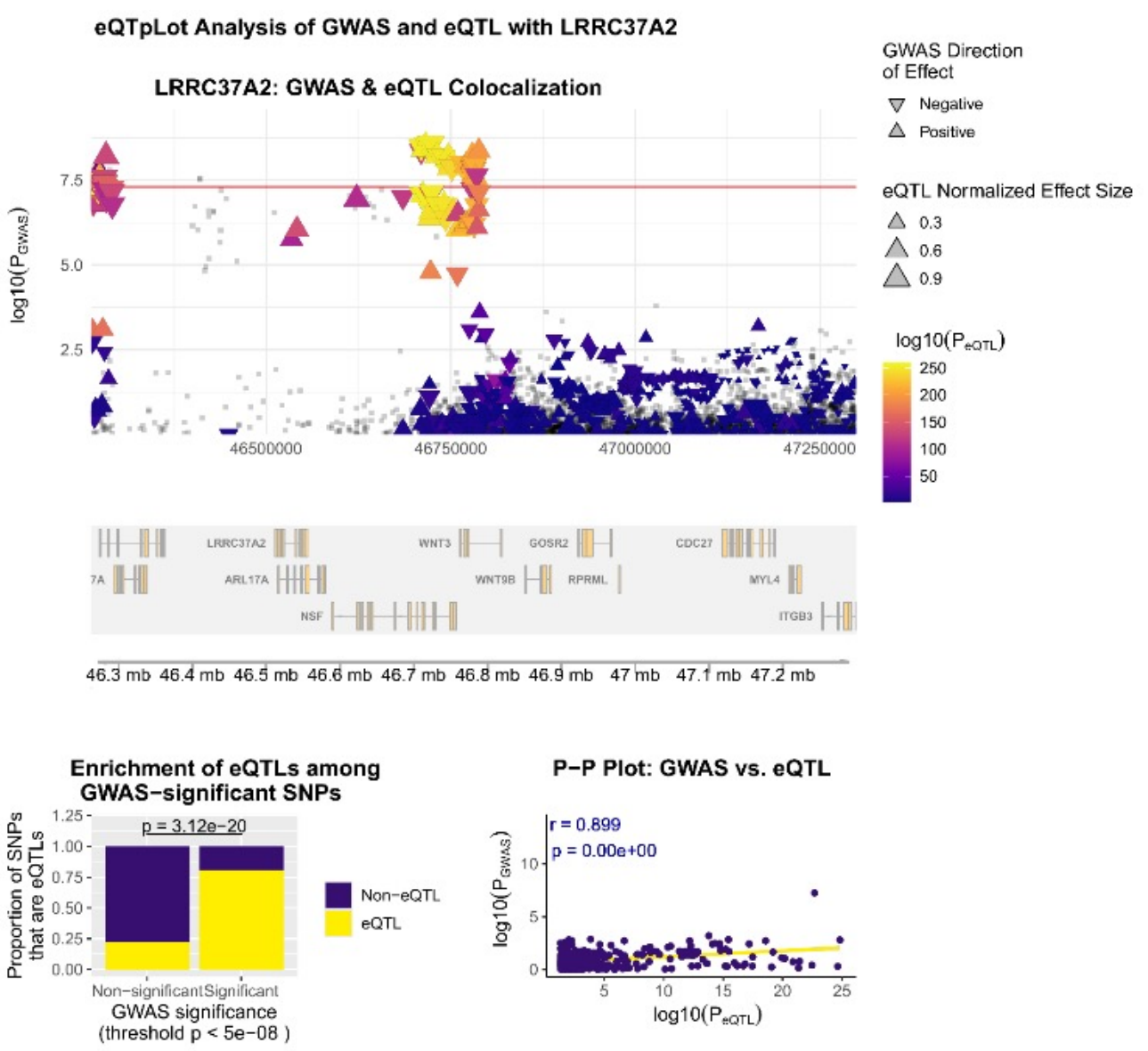
eQTpLot Analysis of GWAS and eQTL with LRRC37A2
LRRC37A2: GWAS & eQTL Colocalization
GWAS Direction of Effect
Negative
Positive
eQTL Normalized Effect Size
log10(P_GWAS)
log10(P_eQTL)
Enrichment of eQTLs among GWAS-significant SNPs
p = 3.12e-20
Non-eQTL
eQTL
Proportion of SNPs that are eQTLs
GWAS significance (threshold p < 5e-08)
P-P Plot: GWAS vs. eQTL
r = 0.899
p = 0.00e+00

f

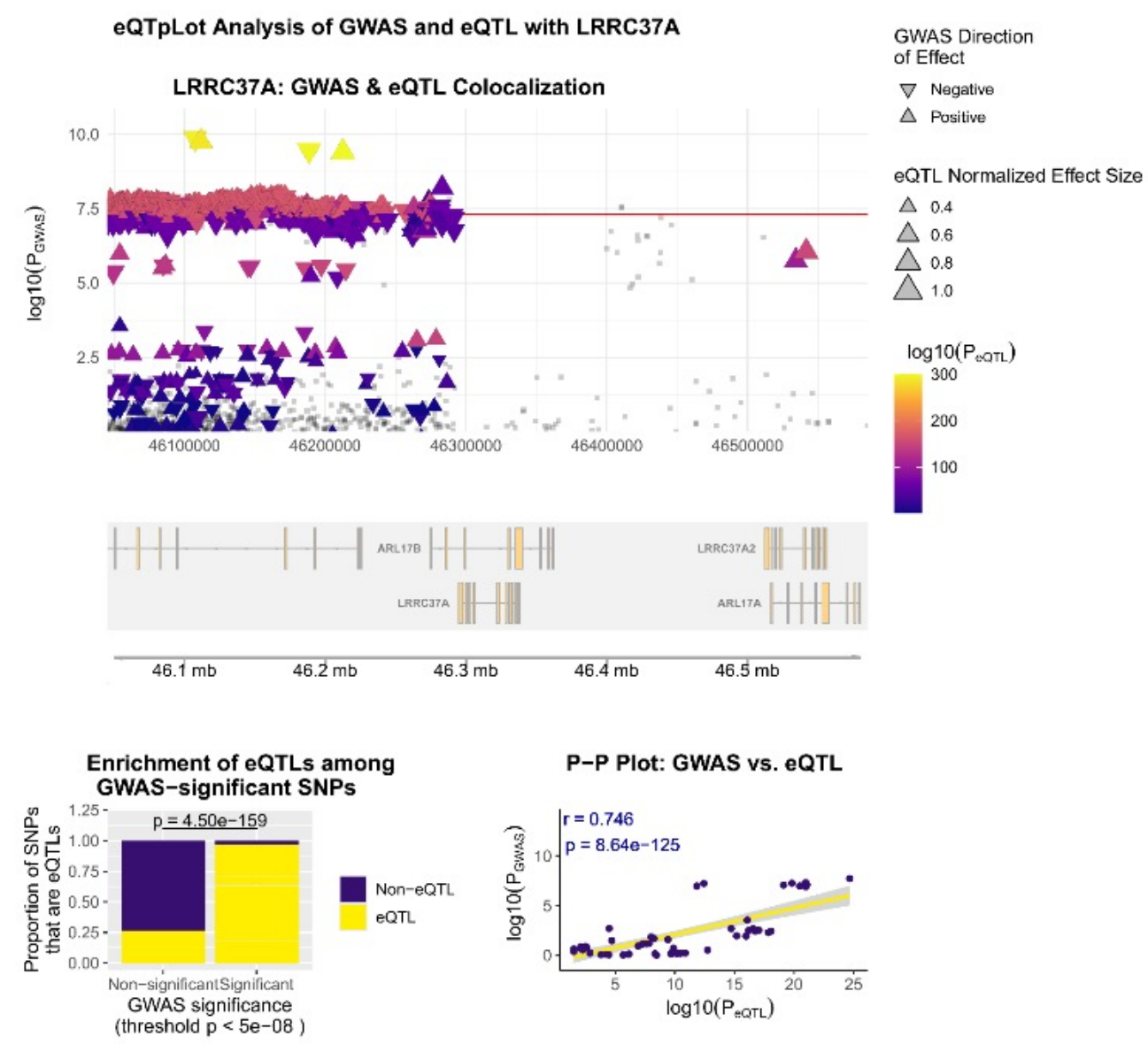
eQTpLot Analysis of GWAS and eQTL with LRRC37A
LRRC37A: GWAS & eQTL Colocalization
GWAS Direction of Effect
Negative
Positive
eQTL Normalized Effect Size
log10(P_GWAS)
log10(P_eQTL)
Enrichment of eQTLs among GWAS-significant SNPs
p = 4.50e-159
Non-eQTL
eQTL
Proportion of SNPs that are eQTLs
GWAS significance (threshold p < 5e-08)
P-P Plot: GWAS vs. eQTL
r = 0.746
p = 8.64e-125

g

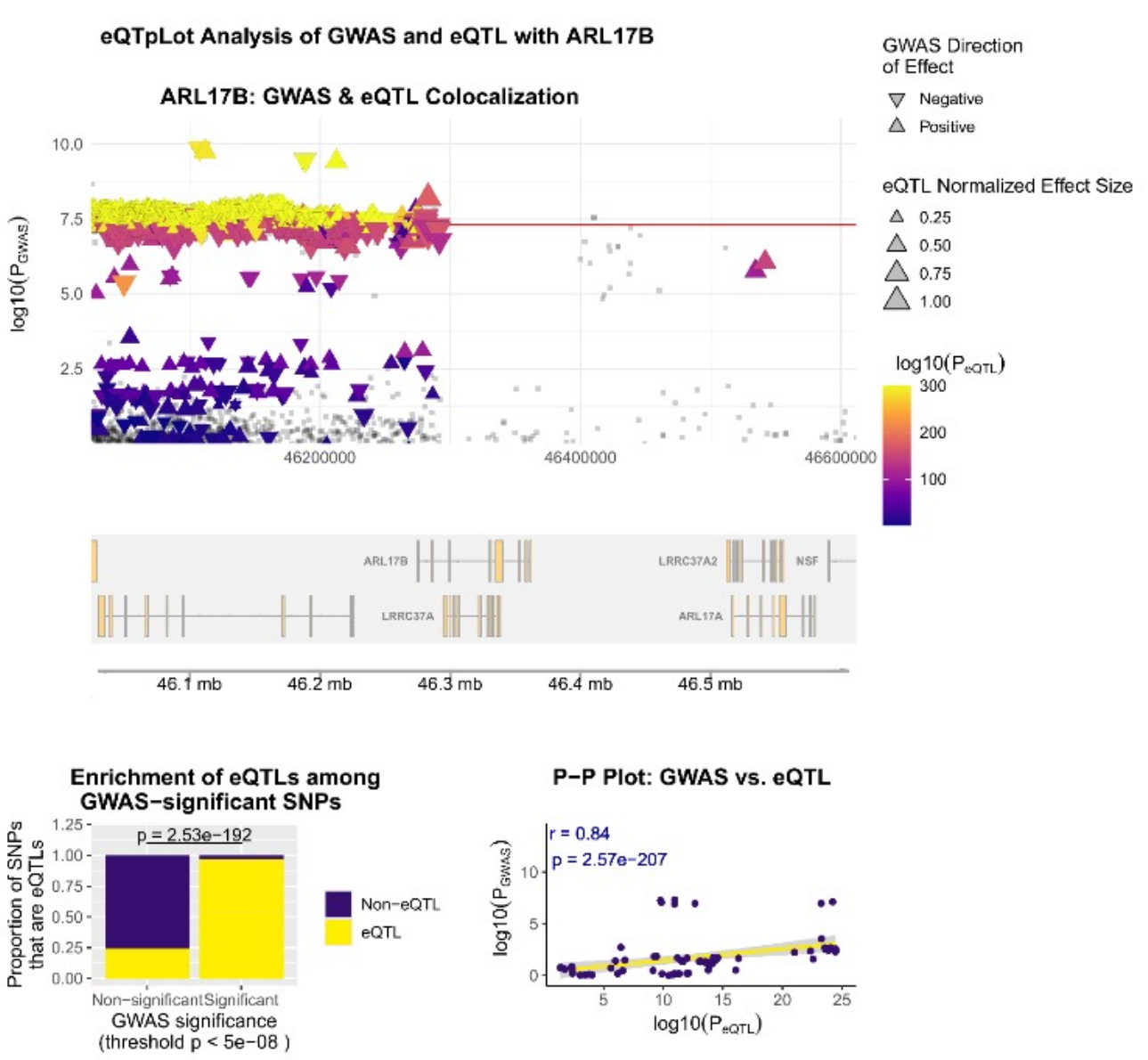
eQTpLot Analysis of GWAS and eQTL with ARL17B
ARL17B: GWAS & eQTL Colocalization
GWAS Direction of Effect
Negative
Positive
eQTL Normalized Effect Size
log10(P_GWAS)
log10(P_eQTL)
Enrichment of eQTLs among GWAS-significant SNPs
p = 2.53e-192
Non-eQTL
eQTL
Proportion of SNPs that are eQTLs
GWAS significance (threshold p < 5e-08)
P-P Plot: GWAS vs. eQTL
r = 0.84
p = 2.57e-207

h

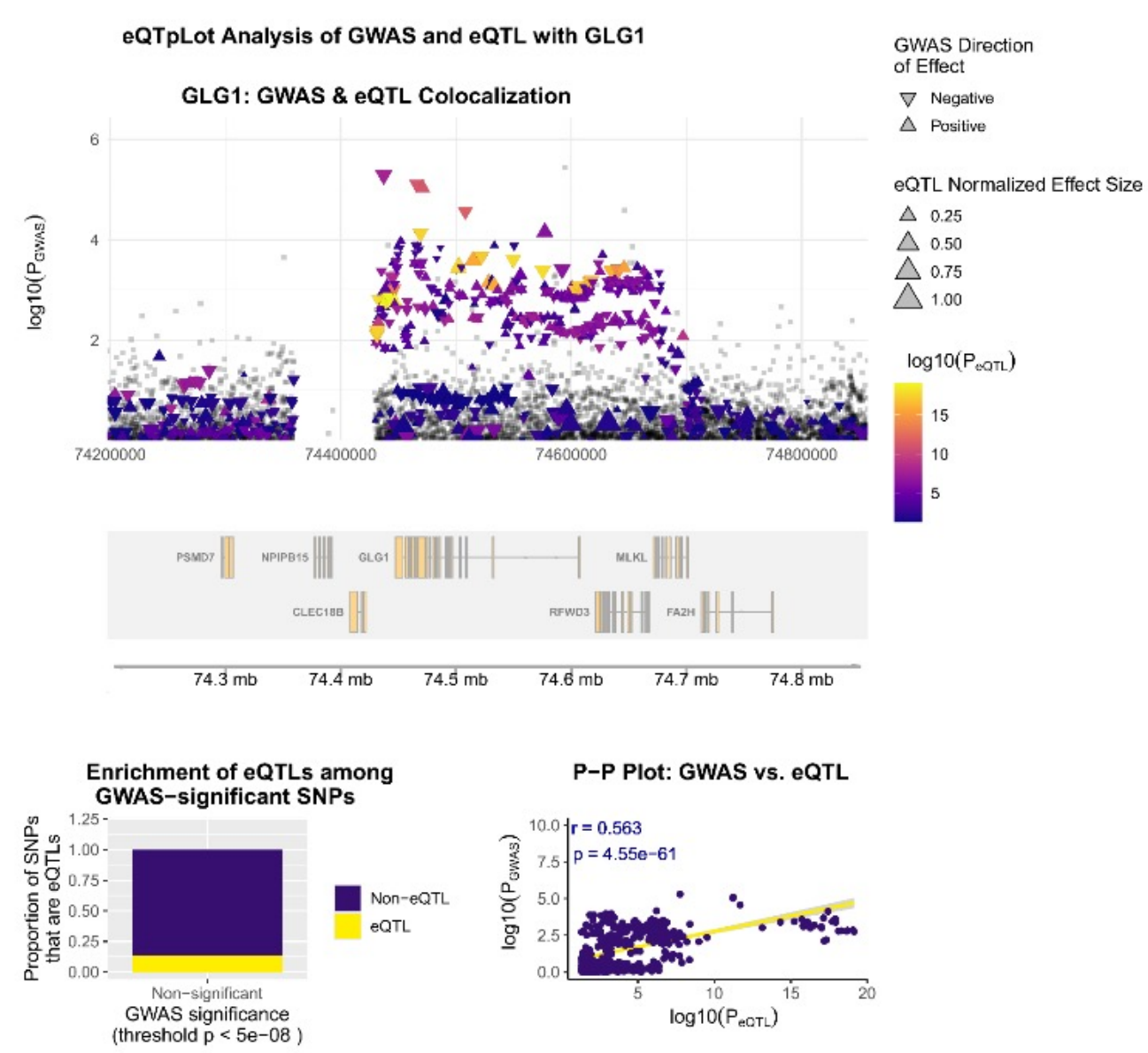
eQTpLot Analysis of GWAS and eQTL with GLG1
GLG1: GWAS & eQTL Colocalization
GWAS Direction of Effect
Negative
Positive
eQTL Normalized Effect Size
log10(P_GWAS)
log10(P_eQTL)
Enrichment of eQTLs among GWAS-significant SNPs
Non-eQTL
eQTL
Proportion of SNPs that are eQTLs
GWAS significance (threshold p < 5e-08)
P-P Plot: GWAS vs. eQTL
r = 0.563
p = 4.55e-61

Supplemental Fig. 8

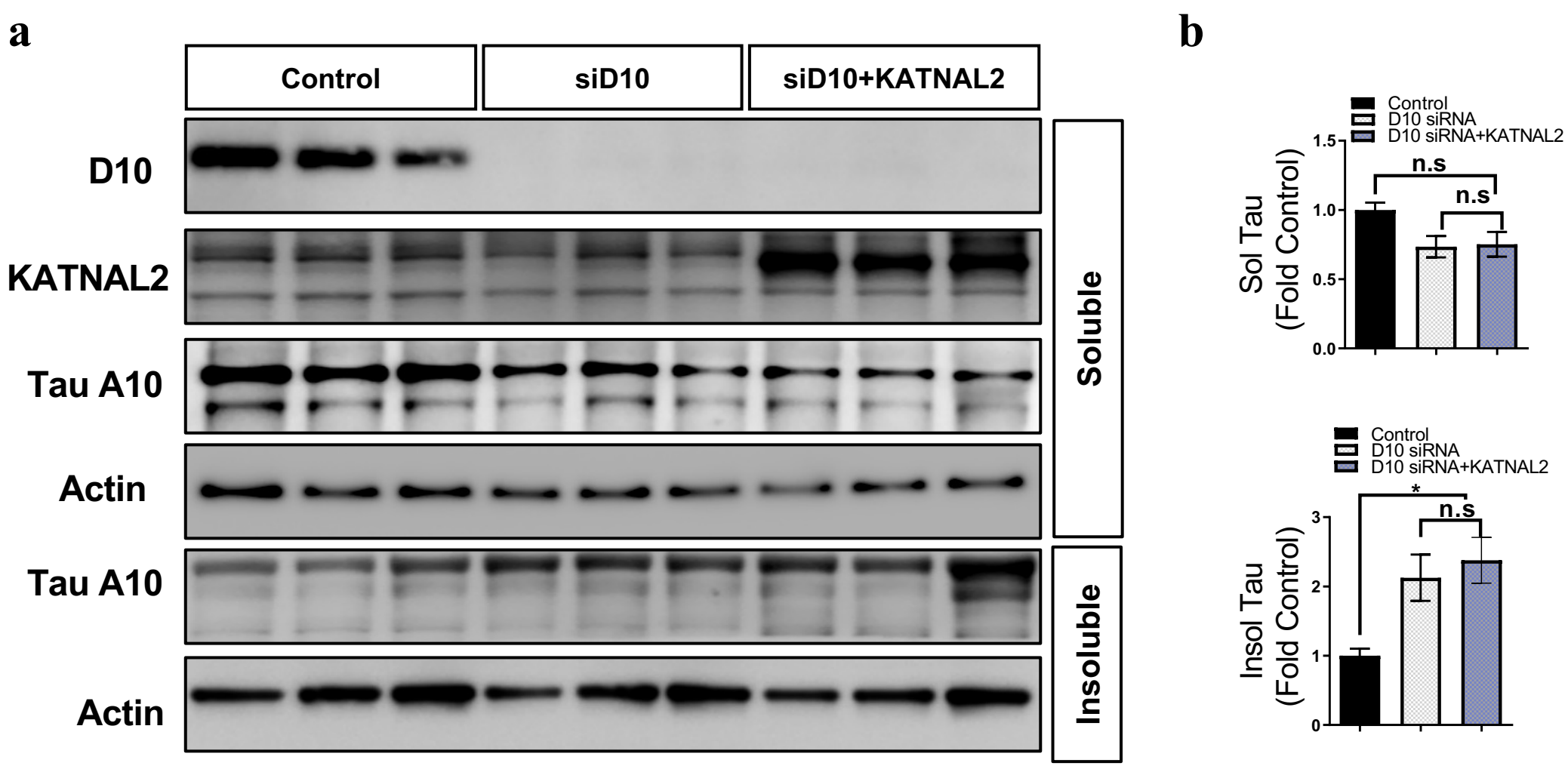

Supplement: Supplementary file 2 [file ADVS-13-e76205-s004.pdf]
