# Supplementary material for: CHCHD10 Mitigates Alzheimer's Disease‐Related Phenotypes in Association With Epigenetic Remodeling in Directly Reprogrammed Neurons
Source: Adv Sci (Weinh). 2026 Jun 25:e76205. Online ahead of print. doi: 10.1002/advs.76205 (PMC13337126; doi:10.1002/advs.76205)
Supplement: Supplementary file 1 — Supporting File 1: advs76205‐sup‐0001‐SuppMat.pdf. [file ADVS-9999-e76205-s001.pdf]

Supplemental Figures

Figure S1: Frequency of significant DMRs on each chromosome and CHCHD10 in tau pathology. (a) Stacked bar graph showing frequency of significant DMRs (p-value < 0.05) on each chromosome. AD vs. NC represented by the blue bar and AD + CHCHD10 vs. AD represented by the red bar. (**b, c**) RIPA-soluble and -insoluble pTau231 levels from M17-APP stable cells transfected with control vector and p3x-Flag-CHCHD10 (n=4, t-test *p-value < 0.05, **p-value < 0.01). (**d, e**) RIPA-soluble and -insoluble tau and pTau231 levels from M17-APP stable cells transfected with control siRNA and CHCHD10 siRNA (n=3, t-test *p-value < 0.05, n.s: not significant).

Figure S2: AD vs. NC neuron DMRs with brain DMRs. (**a**) Miami plot of AD vs. NC DMRs from Brain MTG, Banner Sun Health Institute, and Neuron, primary fibroblast converted neurons. Fisher exact test p-value of genes within 50kb of a significant (p-value < 0.05) DMR between the two datasets is 3.789×10^{-4}. (**b**) Miami plot of AD vs. NC DMRs from Brain PFC, ROSMAP, and Neuron, primary fibroblast converted neurons. Fisher exact test p-value of genes within 100kb of a significant (p-value < 0.05) DMR between the two datasets is 1.002×10^{-2}.

Figure S3: AD vs. NC brain and neuron overlap with AD + CHCHD10 vs. AD neuron DMRs. (**a**) Layered Manhattan plot of AD vs. NC Brain MTG, AD vs. NC Neuron, and AD + CHCHD10 vs. AD Neuron DMRs. Fisher exact test p-value of genes within 50kb of a significant (p-value < 0.05) DMR between the AD vs. NC Brain MTG and AD + CHCH10 vs. AD Neuron datasets is 8.85×10^{-2}. (**b**) Layered Manhattan plot

of AD vs. NC Brain PFC, AD vs. NC Neuron, and AD + CHCHD10 vs. AD Neuron DMRs. Fisher exact test p-value of genes within 100kp of a significant (p-value < 0.05) DMR between the AD vs. NC Brain PFC and AD + CHCH10 vs. AD Neuron datasets is 3.37×10^{-2}.

Figure S4: CHCHD10 subcellular localization, protein interactions, and co-methylation network analysis. (**a**) Cytoplasmic and nuclear fractions of M17-APP cells transfected with vector control and p3x-Flag-CHCHD10 subjected to immunoblotting for Flag-CHCHD10, TOMM20, and H3 proteins. (**b**) Immunofluorescence staining of endogenous CHCHD10 in AD fibroblast-reprogrammed neurons, demonstrating partial nuclear localization. (**c**) Top-ranked CHCHD10-interacting proteins identified by immunoprecipitation-based proteomics from cortical brain tissue of 8-month-old APP/PS1;CHCHD10 mice. Proteins detected in CHCHD10-IP'd samples were ranked by log2 enrichment, calculated from the mean LFQ intensity across CHCHD10 IP replicates relative to the bead-only background control. Proteins shown were classified as strong candidates or high-confidence candidates. Strong candidates were detected in ≥2 of 3 CHCHD10 IP replicates and were undetected in the control lane. High-confidence candidates additionally showed spectral counts ≥10 in all three CHCHD10 IP replicates. Bar colors indicate candidate confidence level, and selected proteins are labeled. (CHCHD10_IP_Proteomics_Raw_Processed_Data.xlsx). (**d**) Representative images of proximity ligation assay (PLA) detecting Flag-CHCHD10 and endogenous OGT in 8-month-old APP/PS1; CHCHD10 mouse brain sections. Red puncta indicate PLA signal; nuclei are stained with DAPI (blue). Minimal signal was observed in

negative control sections, whereas increased PLA signal was detected in Flag-CHCHD10-expressing cells. In negative control, primary antibodies were not added. (**e, f**) PLA detecting interaction between Flag-CHCHD10 and OGT in HT22 cells under control and p3x-Flag-CHCHD10 overexpression conditions. Quantification of PLA puncta intensity (n = 4; t-test, **p < 0.01). (**g**) Co-Methylation Network based on a similarity threshold of 0.9. Node degree represents the number of connections, similarly methylated datapoints, each node has. Betweenness is a measure of how often each node appears on the shortest paths between other nodes. Significant (p-value < 0.05) DMRs in the AD + CHCHD10 vs. AD aggregate dataset annotated to the gene the methylation change was within, or if not within a gene, the closest downstream gene.

Figure S5: Methylation windows. (**a**) Functional annotation of DMRs at the KATNAL2 locus, showing absolute methylation differences and overlap with candidate cis-regulatory elements (cCREs), including promoter, proximal enhancer, distal enhancer, and CTCF-associated regions. Insets highlight representative regions with overlapping DMRs and regulatory elements. (**b–d**) Lollipop plots of significant DMRs (p-value < 0.05) for ZNG1E, SHROOM2, and chr19:1-1,021,123.

Figure S6: Additional Linear Models. (**a**) Linear model using AFR eQTL data: p-value = 0.45. (**b**) Linear model using EAS eQTL data: p-value = 1.4×10^{-5}.

Figure S7: LOI colocalization. (**a**) TAOK2 LOI eQTpLot: 250 kb window, P-P plot r =0.701, P-P plot p-value = 1.81×10^{-35}, Fisher exact test enrichment p-value = 2.07 x

10^{-105}. (**b**) TBX6 LOI eQTpLot: 250 kb window, P-P plot r =0.494, P-P plot p-value = 3.89×10^{-28}, Fisher exact test enrichment p-value = 1.02×10^{-65}. (**c**) KANSL1 LOI eQTpLot: 250 kb window, P-P plot r =0.862. (**d**) WNT3 LOI eQTpLot: 250 kb window, P-P plot r =0.219, P-P plot p-value = 4.54×10^{-9}, Fisher exact test enrichment p-value = 5.93×10^{-18}. (**e**) LRRC37A2 LOI eQTpLot: 250 kb window, P-P plot r =0.899, Fisher exact test enrichment p-value = 3.12×10^{-20}. (**f**) LRRC37A LOI eQTpLot: 250 kb window, P-P plot r =0.746, P-P plot p-value = 8.64×10^{-125}, Fisher exact test enrichment p-value = 4.50×10^{-159}. (**g**) ARL17B LOI eQTpLot: 250 kb window, P-P plot r =0.84, P-P plot p-value = 2.57×10^{-207}, Fisher exact test enrichment p-value = 2.53×10^{-192}. (**h**) GLG1 LOI eQTpLot: 250 kb window, P-P plot r =0.563, P-P plot p-value = 4.55×10^{-61}.

Figure S8: CHCHD10 and KATNAL2 in tau pathology. (**a, b**) RIPA-soluble and -insoluble Tau levels from M17-APP stable cells transfected with control and CHCHD10 siRNA with or without KATNAL2 (n=3, ANOVA p-values = 0.0272, unpaired t-test *p-value < 0.05, n.s: not significant).

Supplemental Tables

Table S1: Differentially methylated regions (DMRs) identified in CpG islands in AD versus NC neurons.

Table S2: Differentially methylated regions (DMRs) identified in promoter regions in AD versus NC neurons.

Table S3: Differentially methylated regions (DMRs) identified in CpG islands in AD+CHCHD10 versus AD neurons.

Table S4: Differentially methylated regions (DMRs) identified in promoter regions in AD+CHCHD10 versus AD neurons.

Table S5: Co-methylation network analysis results of significant DMR-associated genes.

Table S6: Gene Ontology molecular function enrichment analysis of DMR-associated genes in AD+CHCHD10 versus AD neurons.

Table S7: Gene Ontology molecular function enrichment analysis of DMR-associated genes in AD versus NC neurons.

Table S8: Human Phenotype Ontology enrichment analysis of DMR-associated genes in AD+CHCHD10 versus AD neurons.

Table S9: Human Phenotype Ontology enrichment analysis of DMR-associated genes in AD versus NC neurons.

Table S10: Bayesian colocalization analysis between AD GWAS loci and cortex-specific cis-eQTL signals.

Table S11: Linear regression analysis between DMR methylation changes and eQTL effect sizes in the European population.

Table S12: Linear regression analysis between DMR methylation changes and eQTL effect sizes in the African population.

Table S13: Linear regression analysis between DMR methylation changes and eQTL effect sizes in the East Asian population.
