# Supplementary material for: CHCHD10 Mitigates Alzheimer's Disease‐Related Phenotypes in Association With Epigenetic Remodeling in Directly Reprogrammed Neurons
Source: Adv Sci (Weinh). 2026 Jun 25:e76205. Online ahead of print. doi: 10.1002/advs.76205 (PMC13337126; doi:10.1002/advs.76205)

Supplemental Fig. 1

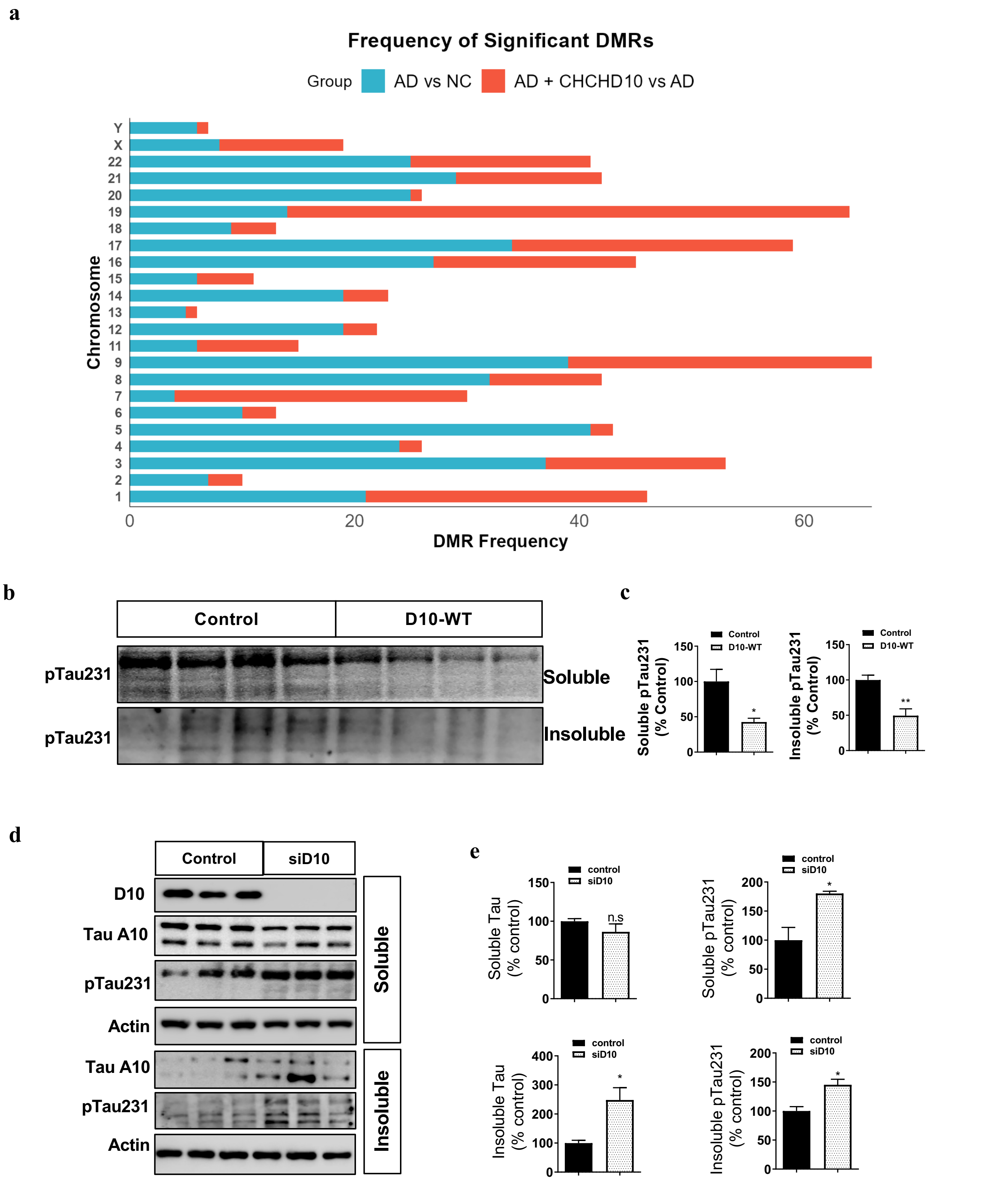

Supplemental Fig.2

a

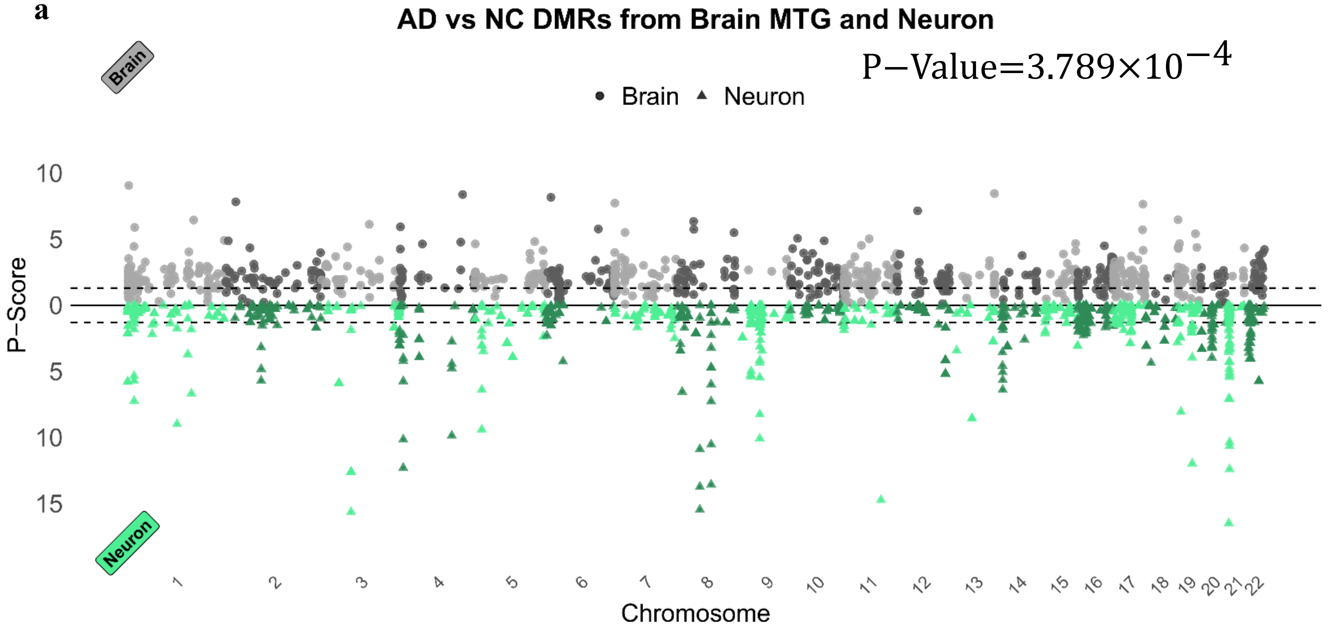

b

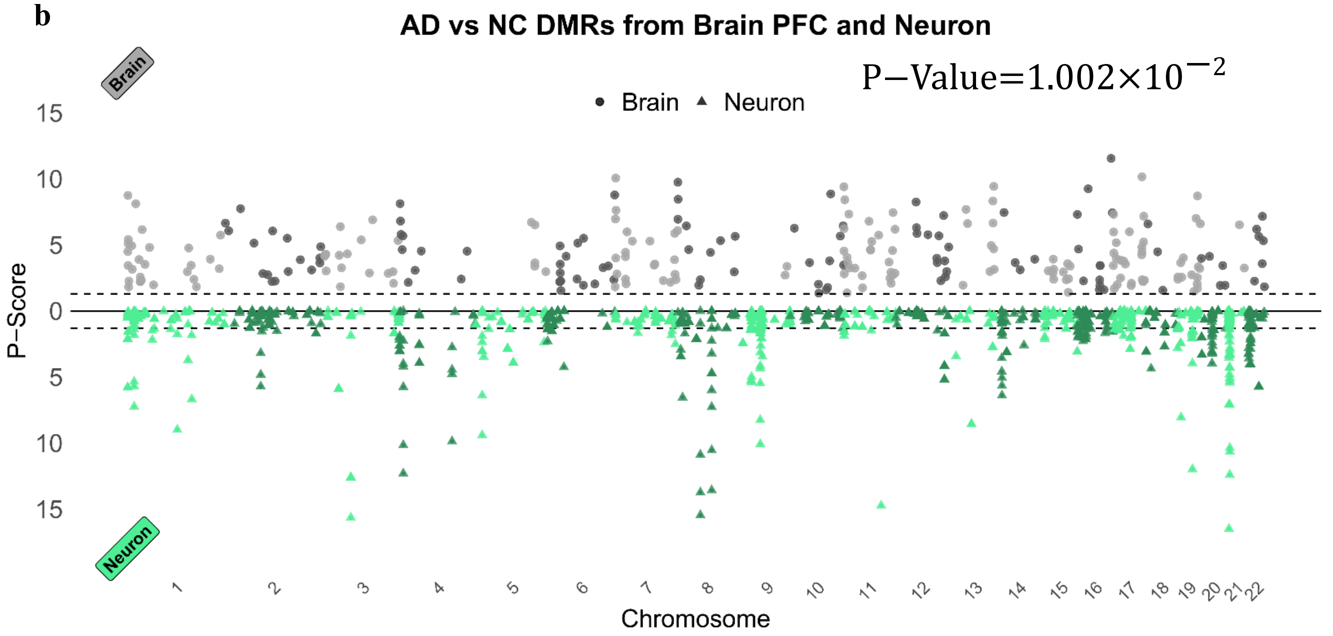

Supplemental Fig.3

a

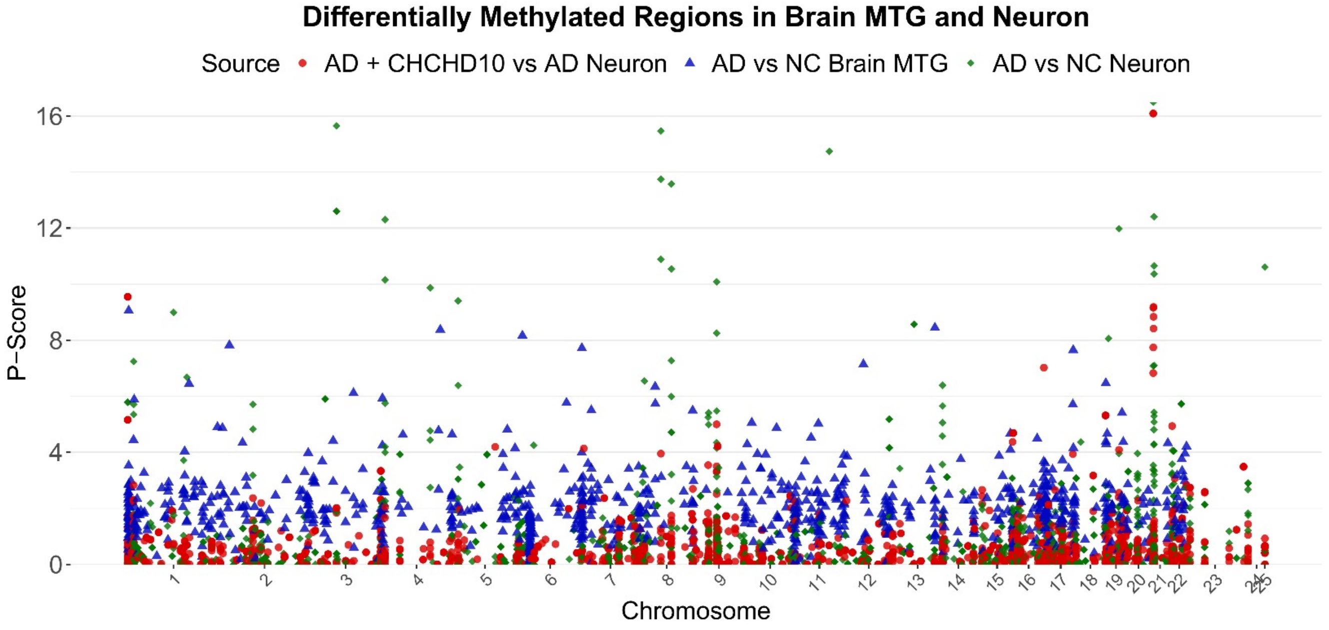

b

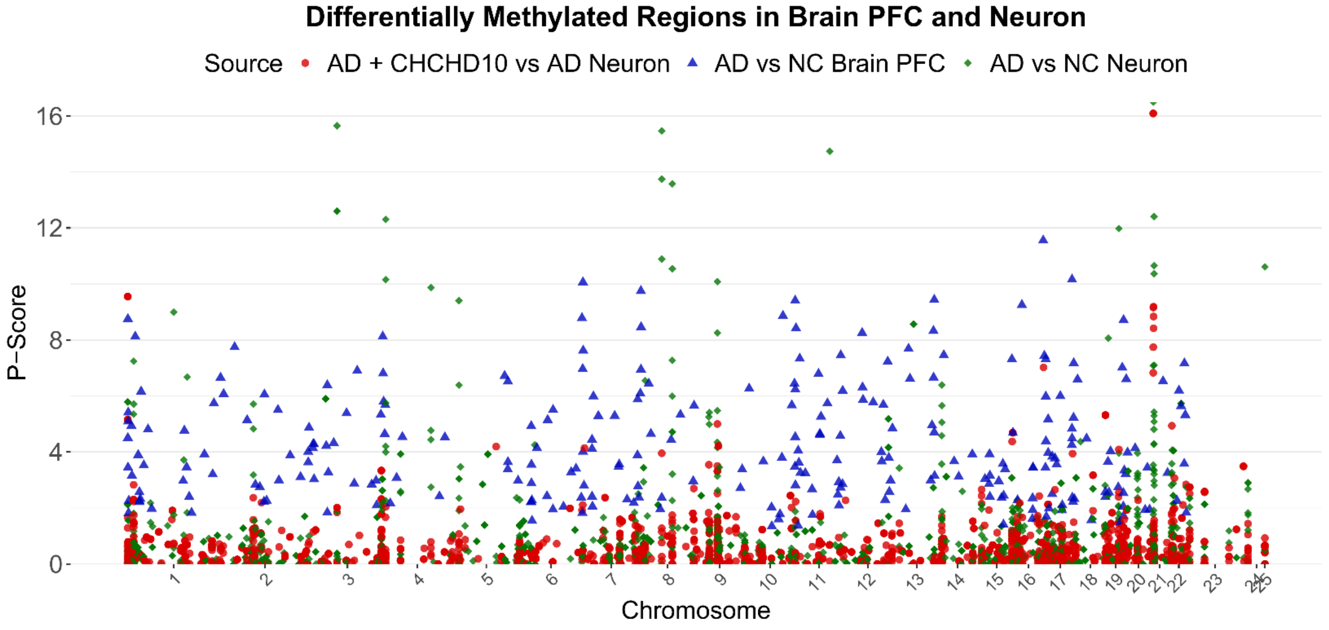

Supplemental Fig. 4

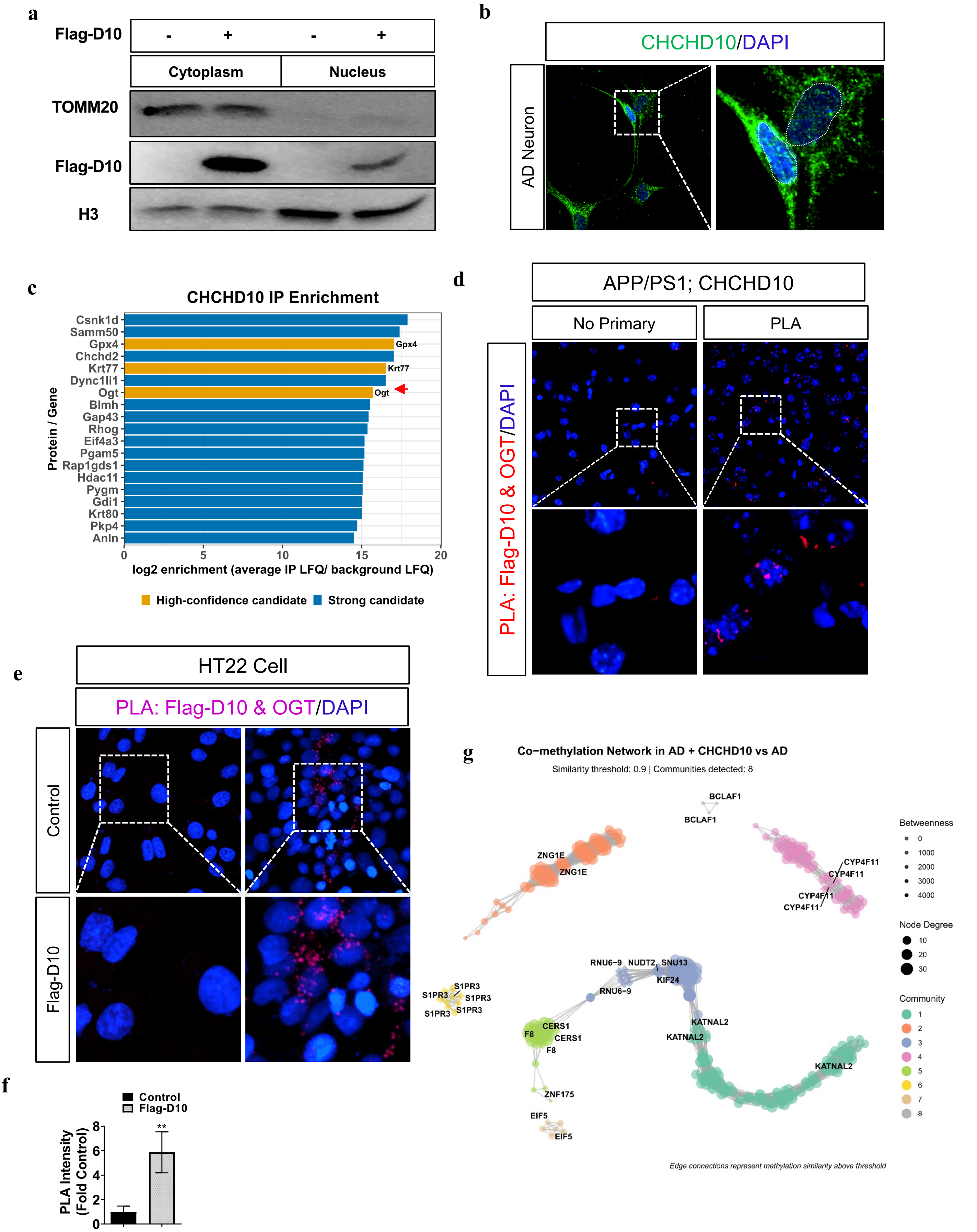

Supplemental Fig. 5

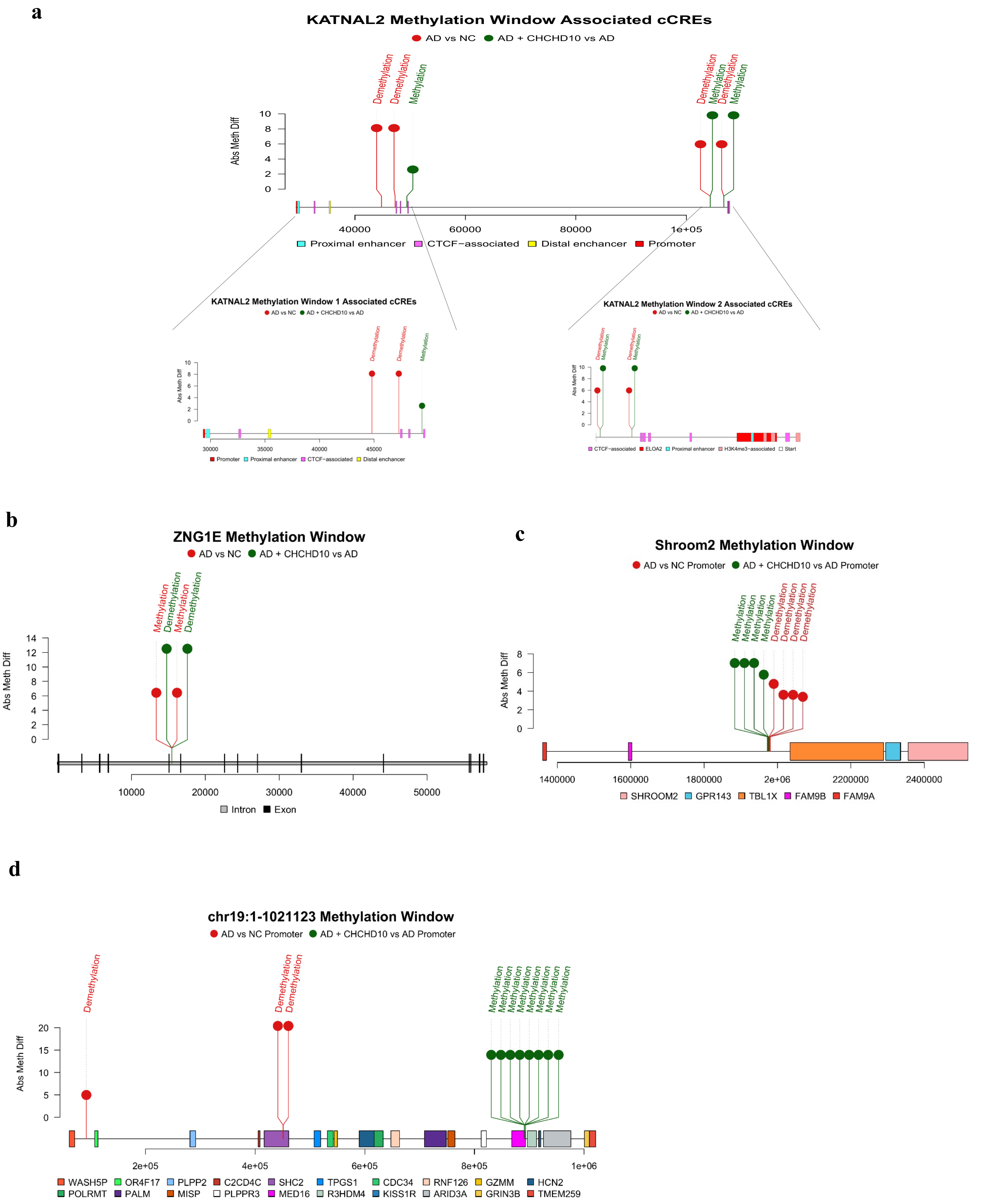

Supplemental Fig. 6

a

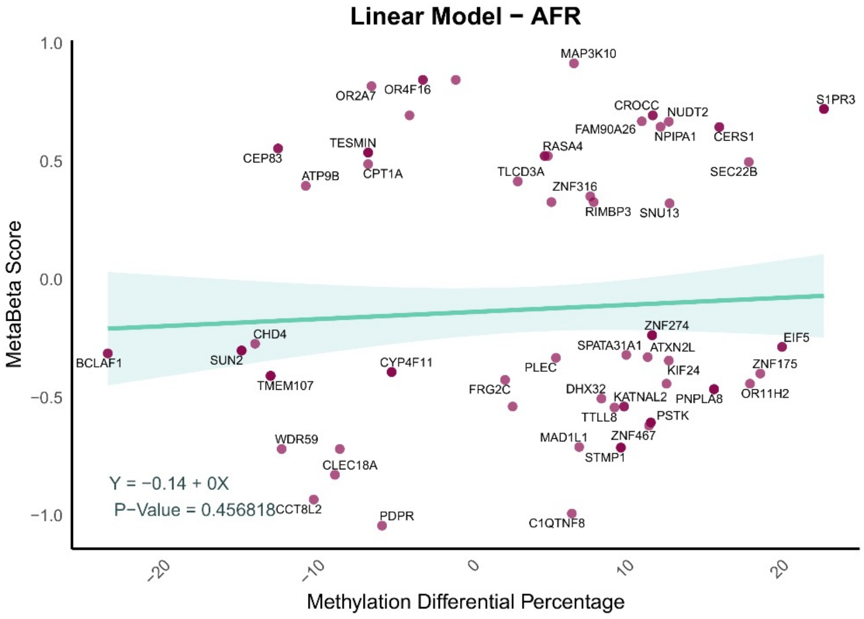

b

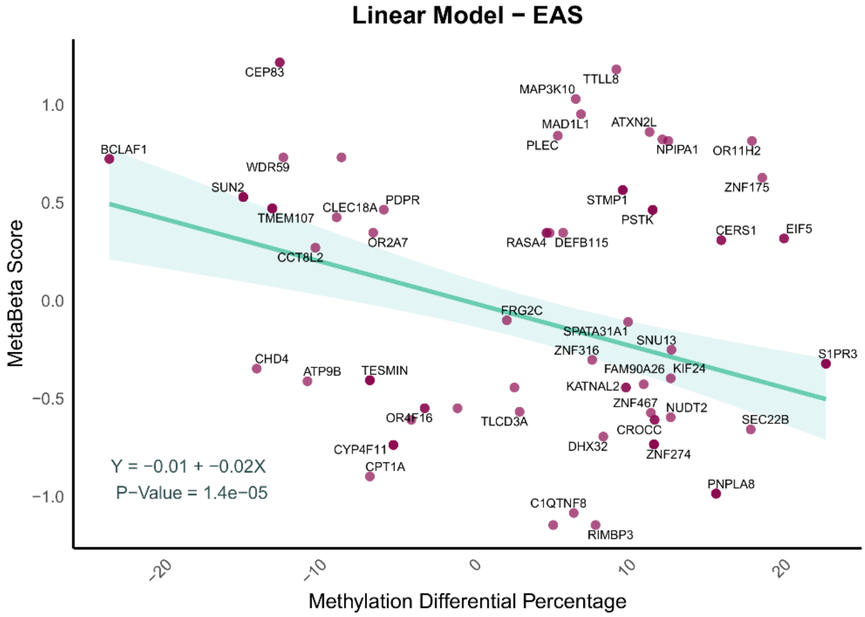

Supplemental Fig. 7

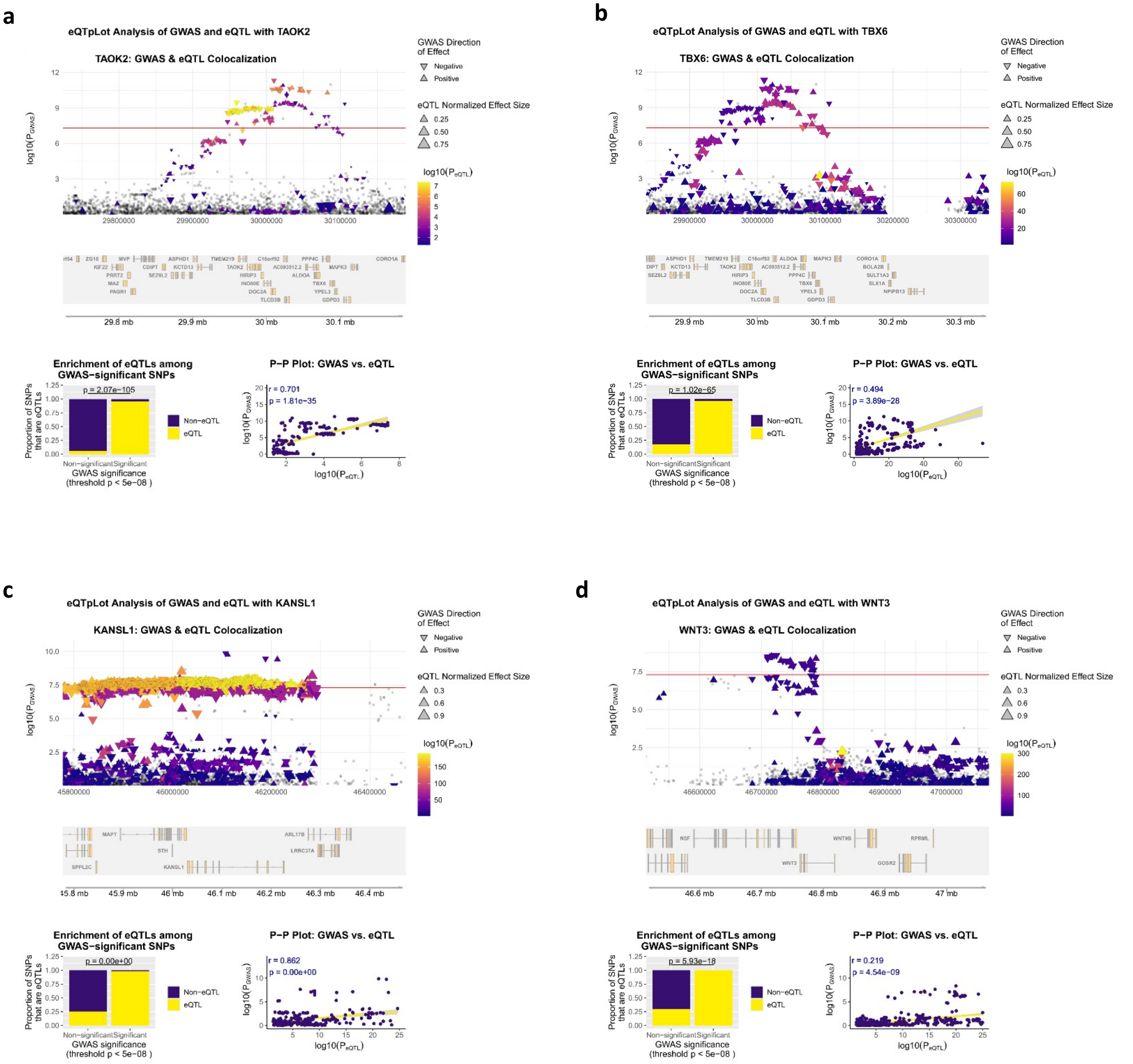

Supplemental Fig. 7 cont.

e

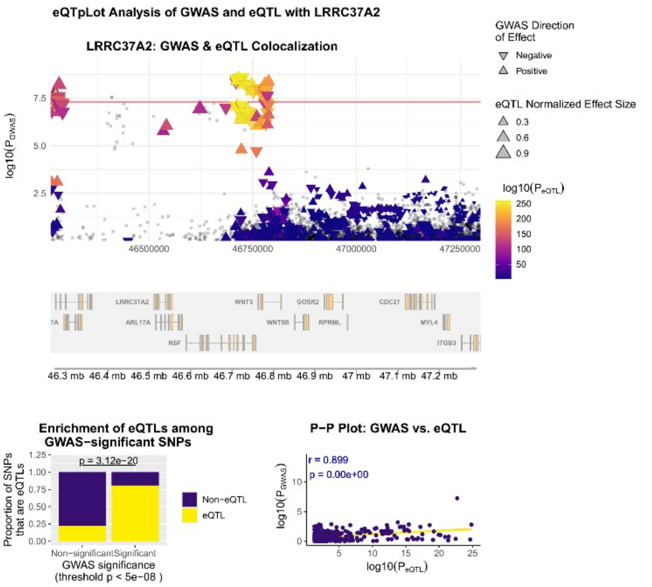

f

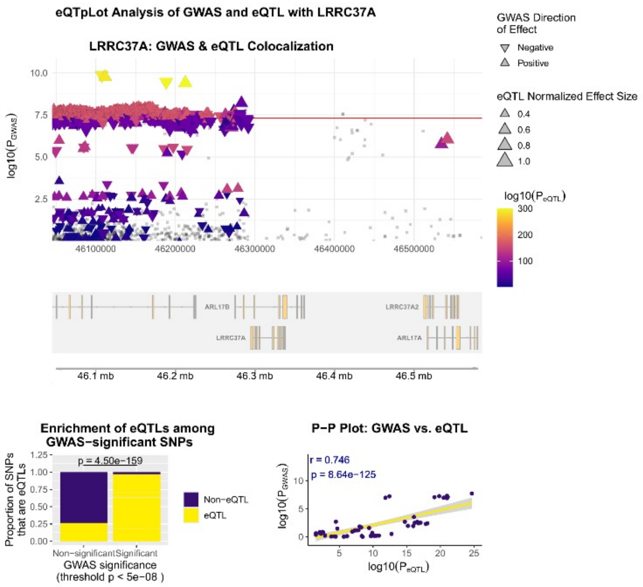

g

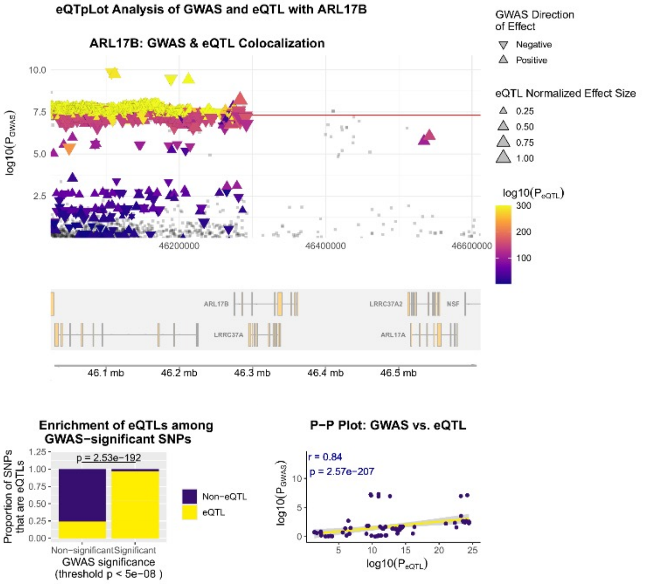

h

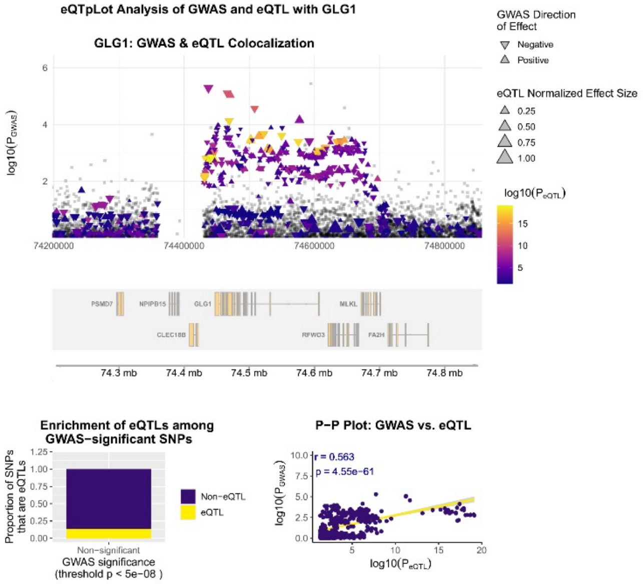

Supplemental Fig. 8

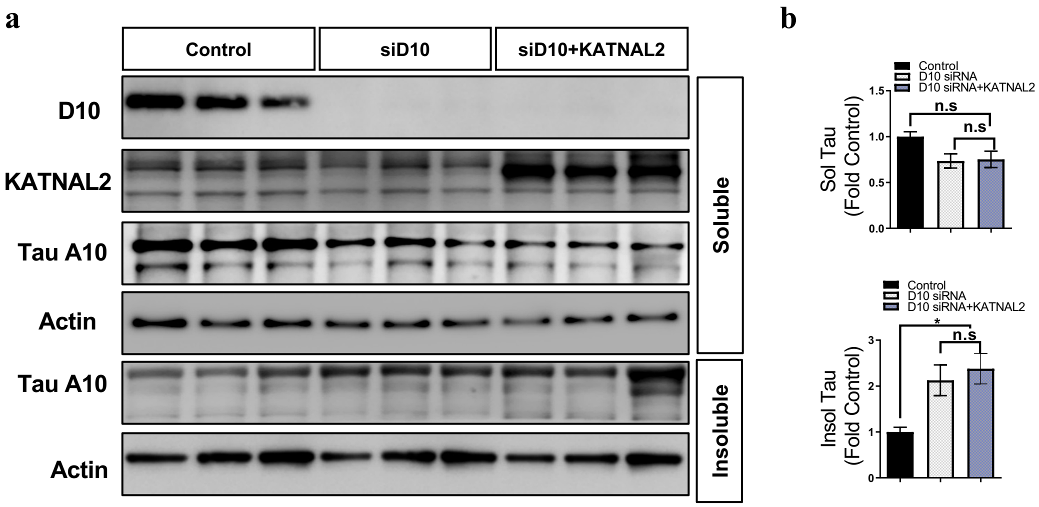

Supplement: Supplementary file 2 — Supporting File 2: advs76205‐sup‐0002‐SupplementalFigures.pdf. [file ADVS-9999-e76205-s003.pdf]
